# In vitro methodology for medical device material thrombogenicity assessments: A use condition and bioanalytical proof‐of‐concept approach

**DOI:** 10.1002/jbm.b.34705

**Published:** 2020-09-14

**Authors:** Michael F. Wolf, Gaurav Girdhar, Arielle A. Anderson, Samantha R. Ubl, Sinduja Thinamany, Hannah N. Jeffers, Courtney E. DeRusha, Jenny Rodriguez‐Fernandez, Sebastian Hoffmann, Carrie A. Strief

**Affiliations:** ^1^ Core Technologies Medtronic Minneapolis Minnesota USA; ^2^ Core Technologies seh consulting + services Paderborn Germany

**Keywords:** biocompatibility, coagulation, in vitro alternative to animal testing, medical devices, thrombosis

## Abstract

Device manufacturers and regulatory agencies currently utilize expensive and often inconclusive in vivo vascular implant models to assess implant material thrombogenicity. We report an in vitro thrombogenicity assessment methodology where test materials (polyethylene, Elasthane™ 80A polyurethane, Pebax®), alongside positive (borosilicate glass) and negative (no material) controls, were exposed to fresh human blood, with attention to common blood‐contact use conditions and the variables: material (M), material surface modification (SM) with heparin, model (Mo), time (T), blood donor (D), exposure ratio (ER; cm^2^ material/ml blood), heparin anticoagulation (H), and blood draw/fill technique (DT). Two models were used: (1) a gentle‐agitation test tube model and (2) a pulsatile flow closed‐loop model. Thrombogenicity measurements included thrombin generation (thrombin‐antithrombin complex [TAT] and human prothrombin fragment F1.2), platelet activation (β‐thromboglobulin), and platelet counts. We report that: (a) thrombogenicity was strongly dependent (*p* < .0001) on M, H, and T, and variably dependent (*p* < .0001 – > .05) on Mo, SM, and D (b) differences between positive control, test, and negative control materials became less pronounced as H increased from 0.6 to 2.0 U/ml, and (c) in vitro‐to‐in vivo case comparisons showed consistency in thrombogenicity rankings on materials classified to be of low, moderate, and high concern. In vitro methods using fresh human blood are therefore scientifically sound and cost effective compared to in vivo methods for screening intravascular materials and devices for thrombogenicity.

## 1 INTRODUCTION

When an intravascular medical device is placed in contact with a patient's circulatory system a variety of reactions are recognized to take place in blood and on the device material surfaces.^1^, ^2^, ^3^ Factors such as surface area of exposure, supplementary anticoagulants, the type of medical device material, and device form can influence the nature and extent of these reactions and potentially influence the level of safety or risk to the patient. Fortunately, the materials used in medical devices come from a select list of high‐performance polymers, metals, ceramics, and biological tissues and have an established history of safe use in humans. Novel devices, which introduce new or unproven materials are increasingly less common, given the costs associated with material qualification processes. As a result, device and material evolution can be protracted and is often achieved through simple or slight changes in materials, material geometry, contact surface chemistry, source (vendor), composition, or manufacturing processes as they are introduced in next generation devices. Within the context of the highly diverse and regulated environment of medical device/material applications, sound material science, toxicology science, and biocompatibility science are required to define device safety and risk.

One of the greatest limitations in the risk assessment process involving intravascular biomaterials is in methods available to assess risk of thrombosis. Even with today's technologies, few standardized in vitro material “thrombogenicity” tests exist,^4^ and existing tests rely on test conditions that are moderately removed from device use conditions (uc). Often initial testing resorts on simple clotting time and platelet counts to measure material thrombogenicity.^5^, ^6^ These metrics represent a single data point in a complex coagulation process and often do not offer a distinction between individual devices and iterative device improvements. Another reason for this discrepancy is the multifactorial nature of the process of thrombosis and the lack of systematic and controlled studies on key variables involved in material thrombogenicity assessment. Such variables include, for example: material, material surface roughness, surface chemistry, overall material/device geometry, species of blood, freshness of blood, subject/donor genetics, anticoagulant type and amount, blood exposure time, and material blood exposure ratio (surface area [cm^2^] of test material to volume of blood [ml] in the test model). This list goes on to include factors such as hematocrit, temperature, presence of an air interface, and an assortment of test model particularities, such as model complexity, blood‐contact surface area of the model itself, gentle mixing versus physiological flow, and so forth. In addition, modern bioanalytical techniques are often antibody‐based and species‐specific and require careful scrutiny for sensitivity to detect statistically and clinically meaningful differences between positive controls, negative controls, and test materials.

In this proof‐of‐concept report we attempt to address actual use conditions and bioanalytical limitations associated with in vitro thrombogenicity assessment of medical device materials. Our report begins with a series of simple exploratory screening experiments that take a first principles look at some of the key variables that can affect the outcome of an in vitro material thrombogenicity test. To control cost and complexity of these material thrombogenicity exploratory studies (MTESs), investigations were limited to: examination of five different materials; testing all conditions at n = 2; limited exploration on the effect of exposure time and test material exposure ratio (cm^2^/ml blood); blood type and freshness restricted to human blood that was directly‐drawn into the models with resultant instant exposure to test and control materials; experiments conducted using blood from a small pool of healthy male‐only donors (4; age range 35–60 years old); heparin anticoagulant ranging from 0.6–2.0 U/ml; material heparin surface modification without rigorous process optimization; and testing using two simple in vitro models. Measurements for assessing material thrombogenicity included assays for thrombin generation (thrombin‐antithrombin complex (TAT) and prothrombin fragment F1.2 (F1.2) ELISAs) and platelet activation (beta‐thromboglobulin (ßTG) ELISA and platelet counts), as recommended in Reference 7. From the outcome of these screening studies, a simplified use condition approach and scoring scheme for an in vitro assessment of material (M) thrombogenicity is proposed based upon thrombin (T) and platelet (P) activation (A) assays. This “ucMTPA” method was subsequently applied to six medical device/material case studies with the in vitro results compared to in vivo thrombogenicity evaluations on the same or similar devices/materials using common animal models. The latter consisted primarily of the nonanticoagulated venous implant (NAVI) model.^3^, ^7^

## 2 MATERIALS AND METHODS

### 2.1 Control and test materials

Each in vitro MTES utilized the following control and test materials: (1) borosilicate glass (“Glass”; the positive control; Schott‐Rohrglas GmbH), (2) high‐density polyethylene (PE; a common reference biomaterial control, Medtronic Santa Rosa, CA), (3) Elasthane™ 80A polyurethane (PEU; a common thermoplastic polyether‐urethane polymer used in medical devices, and a legally marketed comparator device/material (LMCD) per Reference 7; Medtronic Santa Rosa, CA), (4) PEU with CARMEDA® BioActive Surface (designated 'PEU+H'; a commercially available heparin coating used to passivate blood‐contacting medical devices; Medtronic Tijuana), (5) polyether block amide polymer Pebax® ('Pebax'; a common polymer used in vascular catheters; Medtronic Santa Rosa, CA), and (6) No Material (the negative control), that is, the test models themselves, being either standard polyethylene terephthalate (PET) blood test tubes (Becton Dickinson) or polyvinyl chloride (PVC) tubing (Medtronic) closed loops, without test or control material. All test and control materials were nominal 2.0 mm OD solid cylindrical geometry cut into short lengths to fit in test tubes and loops at the specified surface areas, with the exception of the Pebax material, which was derived from a 7 Fr Pebax catheter (tube geometry; 63D [after compounding] with 33.5% by weight Bismuth [(BiO)_2_CO_3_], Teleflex Medical, Plymouth, MN). In the latter case, the open ends were plugged with medical grade silicone adhesive to prevent blood contact in the non‐blood‐contact luminal space (RTV silicone adhesive MED‐1137, NuSil, Carpinteria CA). Based on experience, these five materials were selected with the projected material thrombogenicity of: (i) Glass being highly thrombogenic and generating statistically higher thrombogenicity responses than the other materials and the No Material negative control (ii) PE, PEU, and Pebax presenting intermediate levels of thrombogenicity relative to the glass and No Material controls (iii) PEU+H producing responses less than the PEU material, and (iv) the No Material negative control inducing the lowest thrombogenicity responses. The screening nature aspect of this work drove the exclusion of expensive and time‐consuming clinical‐grade heparin coating process optimization steps designed to maximize coating uniformity and heparin antithrombin bioactivity. Internal experience has shown that without optimization steps, PEUs in general will have an immobilized heparin bioactivity at the lower end of the target therapeutic level of ≥0.1 IU/cm^2^ thrombin deactivation bioactivity test. Thus, there was uncertainty over the extent to which expectation (iii) would be met.

### 2.2 Human blood procurement

All human blood was collected from healthy adult volunteers in accordance with Medtronic policies and with specific informed consent granted by each donor. Institutional Review Board approved protocols were used throughout (Western Institutional Review Board Protocol # 20122029).

### 2.3 Blood exposure test models

#### 2.3.1 *Test tube model*

**FIGURE 1:**
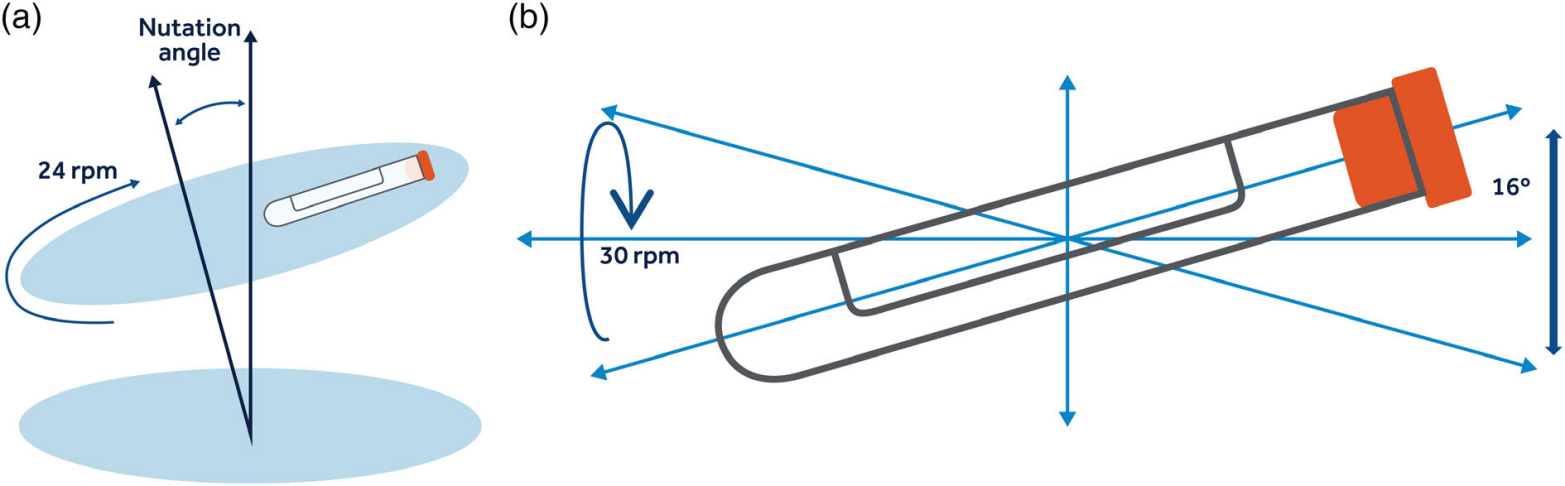
Mixing motion applied in the tube model. Left = Nutation; Right = Combined tilting and rotation

This model utilized standard 13 x 75 mm (BD) Vacutainer tubes (No Additive [Z], Becton, Dickinson and Company, ‘BD’, Franklin Lakes, NJ) as the container for controlled exposure of test and control materials to fresh human blood. These low‐reactivity blood tubes made of PET were individually filled with a specific surface area of test and control materials to give an exposure ratio of 6 to 9 cm^2^/ml whole blood. Heparin anticoagulation was achieved using BD Heparin Lock Flush Solution (10 or 100 USP/ml) diluted appropriately with Plasma‐Lyte A (Injection pH 7.4, Multiple Electrolytes Injection, Type 1 USP, Baxter Healthcare Corporation) to give final concentrations of 0.6, 1.0, or 2.0 U/ml whole blood. The tubes were filled with 3.0 ± 0.2 ml fresh human blood via vacuum filling (VF) or heparinized saline displacement filling (SDF) draw techniques. Upon filling, tubes were placed on either a standard nutating mixer (VWR International; 24 rpm, platform tilt angle: 20° fixed) or a roller‐mixer (Stuart SRT9D; 30 rpm, 16 mm rocking amplitude) for 30–90 mins at 37°C (Roll‐In Incubator, Bellco Biotechnology, Vineland, NJ). See Figure 1. The order of test material exposure to blood in each blood draw was randomized, with each draw including additional separate initial (Bi) and final (Bf) blood samples to monitor draw quality, that is, the extent of blood activation during the draw procedure. The randomization was applied to eliminate any bias during the fill process, such as a slow increase or decrease in blood activation. After blood exposure, blood was withdrawn from each tube into syringes prefilled with CTAD solution (citrate, theophylline, adenosine, and dipyridamole; BD reference no. 367947; 1:10 by volume) and put on ice to arrest any further coagulation and platelet activation, post experiment. 0.5–1.0 ml samples were taken for CBC analysis and remaining blood was centrifuged (2,500 x g, 20 mins) and plasma samples (100–300 μl) were aliquoted into cryotubes and stored at −70°C for subsequent ELISA analyses. Test materials were also gently rinsed with Plasma‐Lyte A to remove nonadherent blood and photographed for visible thrombus on the material surfaces. Consult the Appendix for additional details.

#### 2.3.2 *Dynamic closed‐loop model*

**FIGURE 2:**
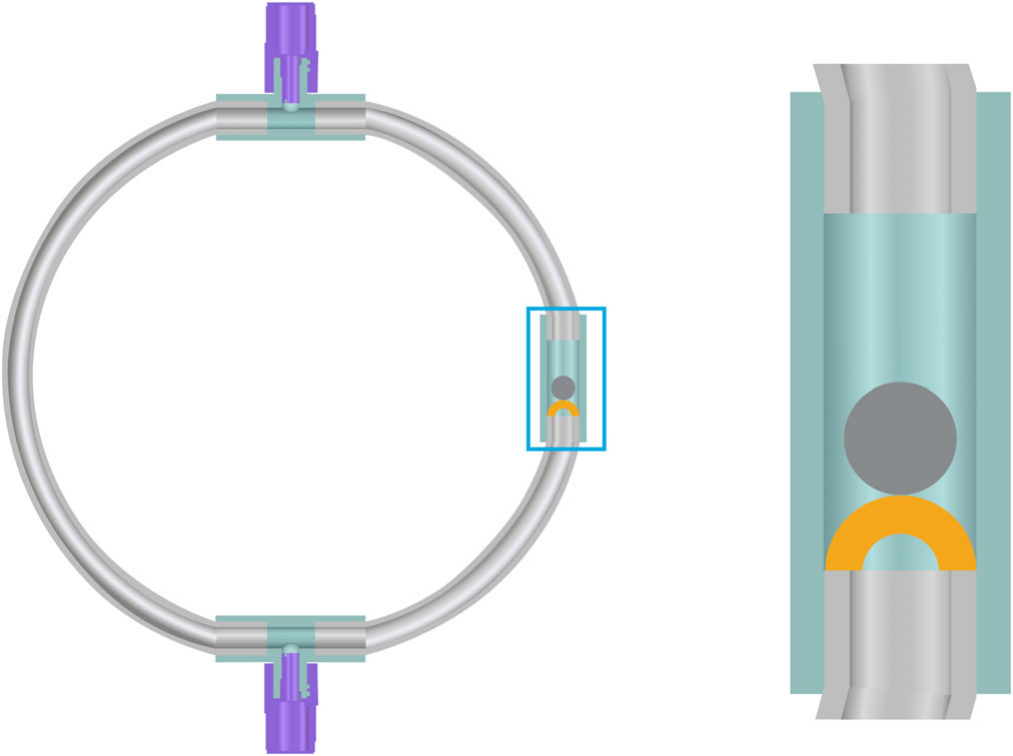
Design of dynamic pulsatile closed loop model (left). The loop contains two injection/withdraw ports (Port 1 [6:00 position] and Port 2 [12:00 position] and a check valve (at 3:00 position; enlargement at right). A repeating (specified) pulse of rotation in the clockwise direction followed by a (specified) pause causes the small check valve to close and open, respectively, and the blood to flow in pulsatile fashion via angular momentum

This more advanced in vitro model employed closed circular loops of PVC tubing as the “test tube” for insertion of test and control materials. These torus‐shaped loops have specific features that allow priming with heparinized saline, no‐air‐exposure blood filling via saline displacement draw technique, and pulsatile flow created by the combination of an integral check valve and applied pulsatile rotational motion (Figure 2). See References 8, 9, 10, 11, 12 and the Appendix for additional details.

#### 2.3.3 *Thrombogenicity assessment using in vitro assays for thrombin generation and platelet activation*

Thrombogenicity measurements consisted of ELISA assays for the key coagulation proteins TAT^13^ and F1.2,^14^ as well as β‐thromboglobulin (βTG), an indicator of platelet activation.^15^ Complete blood counts (CBCs) determined by routine electronic methods (MedTox Laboratory, Minneapolis MN) were used to obtain platelet counts before and after blood‐material exposure. Commercial ELISAs were as follows: TAT (Enzygnost TAT micro, Siemens Healthcare Diagnostics Products GmbH, Marburg, Germany), F1.2 (Enzygnost F1+2 [monoclonal], Siemens Healthcare Diagnostics Products), and β‐TG (Asserchrom β‐TG, Diagnostica Stago, Asnieres, France). The ELISA plates were washed using a Biotek ELx50 Microplate Strip Washer GENEMate AutoWash 50 (Biotek, Winooski, VT) and analyzed using a SpectraMax 384 Plus microplate reader (Molecular Devices, Sunnyvale, CA). ELISA data quality and inclusion criteria consisted of manufacturer‐supplied internal controls falling within target ranges, use of plasma dilution factors that allowed data to fall on the standard curve, and technicians trained to consistently obtain coefficients of variance ≤10%.

### 2.4 MTESs MTES1, MTES2, and MTES3 on essential blood interaction variables

Exploratory “screening” study variables and the study designs are described in Table 1. Completing each study required a series of separate blood donations from each donor (four donors used, designated A through D), allocated by distinct heparin level, and other variables, such as time, model, and so forth. All conditions were tested in duplicate in tubes or loops of test materials exposed to 3.0, 3.2, or 5.0 ml blood per tube/loop (depending on model). All blood donations involved informed consent and required donors to be healthy and drug and aspirin refraining.

**TABLE 1:** Variables and measurements in exploratory studies on in vitro methods to assess material/device thrombogenicity

Material thrombogenicity exploratory study^a^ (MTES)	Main study variables						Measurements	
	Materials	Model^b^	Donor	Exposure ratio (cm^2^/ml)	Exposure time (min)	Heparin level (U/ml)	Coagulation activation	Platelet activation
MTES1	1. Glass 2. PE 3. Pebax 4. PEU 5. PEU+H 6. No Material	Tube (SDF)^1^	A B C	9	30 60 90	0.6 1.0	TAT F1.2	βTG
MTES2	1. Glass 2. PE 3. Pebax 4. PEU 5. PEU + H 6. No Material	Tube (VF) ^2^ Loop (SDF) ^3^	A B	9	60	1.0 2.0	TAT F1.2	βTG
MTES3	1. Glass 2. PE 3. Pebax 4. PEU 5. PEU + H 6. No Material	Tube (VF) ^2^ Loop (SDF) ^3^	A D	6	60	1.0 2.0	TAT F1.2	βTG

*Note*: 1 = 64% blood without air exposure and with nutation mixing, 2 = 94% blood and rocking‐rolling mixing with air sloshing, and 3 = 50% blood without air exposure and with pulsatile flow.

Abbreviations: H, heparin; PE, polyethylene; Pebax, polyether block amide; PEU, polyurethane; PEU+H, polyurethane with a heparin coating; No Material, Tube or Loop model without any test or control materials present; SDF, saline displacement filling; VF, vaccum fill; TAT, thrombin‐antithrombin complex; F1.2, prothrombin fragment F1.2; βTG, β‐thromboglobulin.

^a^ Each study was executed as a full factorial experiment with the following designs, respectively: 6 x 3 x 3 x 2 (material x donor x time x heparin), 6 x 2 x2 x 2 (material x model x donor x heparin), and 6 x 2 x 2 x 2 (material x model x donor x heparin).

^b^ Tube = standard 13 x 75 test tube; Loop = nominal 3/16″ (4.7 mm) ID closed loop; SDF = saline displacement [blood] fill; VF = vacuum [blood] fill.

### 2.5 Case studies on devices or device materials tested for in vitro thrombogenicity

**FIGURE 3:**
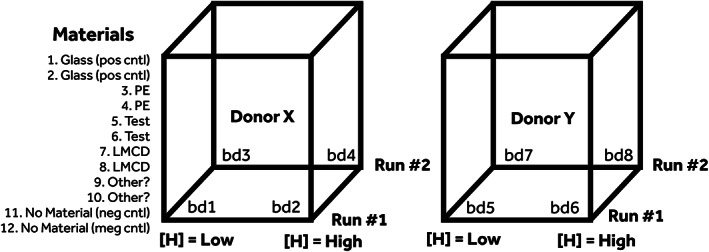
The generic in vitro case study experimental design. This design involves testing a positive control (pos cntl), a No Material negative control (neg cntl), a biomaterial control (PE), and a legally marketed comparator device (LMCD) material alongside the test material (Test) at two heparin levels (where use conditions indicate test devices/materials may be used with and without anticoagulation). It specifies testing with blood from two donors, and potential repeat runs (allowing n = 2 or n = 4) to increase robustness and confidence in results. This gives a Materialx2x2x2 full factorial design that involves 4–8 separate small volume blood draws (bd; i.e., bd1 to bd8)

Six medical device or medical device material cases were evaluated for in vitro thrombogenicity using variable and measurement selection based upon MTES findings. Table 2 provides details on each case study and Figure 3 shows the geometric representation of the generic in vitro study design. The later consisted of using either the tube or loop model along with: blood from two donors (selected from the same pool of MTES donors, and an additional donor E), an exposure ratio of 6.0 cm^2^/ml, heparin anticoagulation at one or two levels (within range of 0.6–2.0 U/ml), blood exposure restricted to 60 minutes, and replication n = 2 (1 run) or 4 (2 runs) per condition. Briefly, Case Study 1 utilized the tube model (VF) with 94% blood to examine a Medtronic vascular catheter test device (Test Device 1) used in cardiac ablation procedures. Here the test catheter contained an experimental silicone oil coating on the blood‐contacting surface and the untreated device served as the LMCD (LMCD 1). Case Study 2 also utilized the tube model (VF) with 94% blood and involved comparing the copolyester polymer Tritan™ (Eastman Chemical Company; LMCD 2) against the same material prepared by Interface Biologics Inc. (IBI, Toronto, Canada). In this case IBI prepared two different loadings of the fluorinated end‐group polymer Endexo® (Test Material 2A and Test Material 2B, containing 4 and 2% Endexo®, respectively). Case Study 3 utilized the loop model (SDF) with 80% blood to compare the Medtronic Pipeline™ aneurysm flow diversion device (LMCD 3) to the same device containing the Shield™ phosphorylcholine hemocompatible surface treatment (Test Device 3). The exposure ratio in this study was four devices per loop (one per quadrant). Case Studies 4 and 5 were nested in the exploratory MTES3 study and utilized both the tube model (VF) with 94% blood and the loop model (SDF) with 50% blood to compare (i) a Medtronic device PEU polymer (LMCD 4) to the same material containing a heparin coating (PEU+H; Test Material 4) and (ii) a Medtronic PEU polymer (LMCD 5) to a Medtronic device Pebax polymer (Test Material 5), respectfully. Case Study 6 utilized a “streamlined” study design that involved testing at only one heparin level and a single thrombogenicity measurement (TAT), using the tube model (VF) with 94% blood. This study compared two different LMCD (LMCD 6A, from Medtronic, and LMCD 6B from a non‐Medtronic manufacturer) to a new Medtronic device manufactured with several new processing steps (Test Device 6). Limited technical information was available for LMCD 6B material, but this material was suspected to have a coating designed to improve hemocompatibility.

**TABLE 2:** Devices or device materials tested for in vitro thrombogenicity using a discrete set of critical test variables

Case study	Main study variables								Study measurements
	Materials (test and control)	Device application	Model^a^	Donor	Exp. ratio cm^2^/ml	Exp. time min	Heparin level U/ml	# of runs	
1	1. Glass 2. PE 3. LMCD 1 4. Test Device 1 5. No Material	Vascular catheter	Tube (VF)	C D	6	60	0.6 1.0	2	TAT βTG Platelets
2	1. Glass 2. PE 3. LMCD 2 4. Test Material 2A 5. Test Material 2B 6. No Material	Polymer‐based vascular device	Tube (VF)	A D	6	60	0.6 1.0	2	TAT βTG Platelets
3	1. LMCD 3 2. Test Device 3 3. No Material	Neurovascular Flow diverter	Loop (SDF)	D E	four devices per loop	60	0.6 1.0	1	TAT βTG Platelets
4	1. Glass 2. PE 3. LMCD 4 4. Test Material 4 5. No Material	Polymer‐based vascular device	Tube (VF) Loop (SDF)	A D	6	60	1.0 2.0	1	TAT F1.2 βTG
5	1. Glass 2. PE 3. LMCD 5 4. Test Material 5 5. No Material	Polymer‐based vascular device	Tube (VF) Loop (SDF)	A D	6	60	1.0 2.0	1	TAT F1.2 βTG
6	1. Glass 2. PE 3. LMCD 6A 4. LMCD 6B 5. Test Device 6 6. No Material	Vascular catheter	Tube (VF)	A D	6	60	0.6	2	TAT only

Abbreviations: LMCD, legally marketed comparator device/material; PE, polyethylene; TAT, thrombin‐antithrombin complex; F1.2, prothrombin fragment F1.2; βTG, β‐thromboglobulin.

^a^ VF, vacuum (blood) filling; SDF, saline displacement (blood) filling.

### 2.6 In vivo methods for device/material testing for thrombogenicity

All animals utilized in this research were cared for according to the policies and principles established by the Animal Welfare Act and the NIH Guide for Care and Use of Laboratory Animals. The test devices/materials listed in Table 2 Case Studies 1, 4, 5, and 6 were tested for in vivo thrombogenicity using the NAVI model.^3^, ^7^ Briefly, the NAVI tests involved inserting a 10–15 cm portion of each device or material, in catheter form, into a vein of a large animal. In these investigations, either a canine or ovine femoral or jugular vein model was used. In all cases, the test material/device was positioned in one vein and the control LMCD material was positioned similarly in the contralateral site. The level of replication differed between these studies, as did the in situ blood exposure duration (1–4 hours). Immediately following euthanasia, the implants were carefully exposed in situ and gently rinsed with buffer to remove nonadherent blood. They were then photographed and assessed for apparent surface thrombus using the scoring scheme shown in Table 3.^3^, ^7^ Thrombogenicity scoring was carried out by one or two qualified pathologists and the scores averaged, when applicable. The in vivo thrombogenicity assessment of the fluorinated polymer material in Case Study 2 received NAVI testing as described in Reference 16. The neurovascular stent devices in Case Study 3 were not tested in a strict NAVI model, but rather received acute in vivo thrombogenicity assessment in both an ex vivo nonhuman primate arteriovenous shunt model^17^ and a New Zealand white rabbit common carotid elastase‐induced aneurysm model.^18^ For demonstrating the in vivo response to a heparin coating, Case 4 utilized a commercially available PEU central venous catheter with a proprietary (Medtronic) heparin coating. Table 4 summarizes the test models and conditions used to study in vivo thrombogenicity.

**TABLE 3:** NAVI scoring scheme^3^, ^7^

Thrombus formation score description	Score
Thrombus nonexistent or minimal and, if present, appears to be associated with implant venotomy site	0
Thrombus minimal, observed to be covering 1–25% of material surfac.	1
Thrombus moderate, observed to be covering 26–50% of material surface	2
Thrombus severe, observed to be covering 51–75% of material surface	3
Thrombus extensive, covers 76–100% of material surface	4

Abbreviation: NAVI, nonanticoagulated venous implant.

**TABLE 4:** In vivo NAVI studies corresponding to in vitro thrombogenicity case studies 1–6 (Table 2)

In vivo case study	Device/material information		Animal model (no anticoagulation)		
	Descriptor^a^	Device/material	Model	Vein	n
1	LMCD 1	Catheter A	Canine	Femoral and jugular	3
	Test Device 1	Catheter A + Si oil			
2	LMCD 2	Catheter B	Ovine	Jugular	12
	Test Device 2	Catheter B + Endexo^b^			
3	LMCD 3	Pipeline stent	Laprine	Subclavian^c^	3
	Test Device 3	Pipeline + shield			
4	LMCD 4	Catheter C	Canine	Femoral	1
	Test Device 4	Catheter C + heparin coating			
5	LCMD 5	Catheter C	Canine	Femoral	4
	Test Device 5	Catheter D			
6	LMCD 6A and 6B	Transseptal dilator E (original) and F (other)	Canine	Femoral/IVC	2
	Test Device 6	New Transseptal dilator G			

Abbreviation: NAVI, nonanticoagulated venous implant.

^a^ LMCD, legally marketed comparator device.

^b^ See in Reference 16.

^c^ See in References 17 and 18, nonanticoagulated arterial implant (NAAI) model.

### 2.7 Statistical analyzes

MTESs were analyzed by ANOVA for main effects and interactions, and by Tukey Kramer HSD for means comparisons, using JMP (SAS Institute Inc., Cary NC) and Design‐Ease (Stat‐ease Inc., Minneapolis MN) software. To normalize data distribution and control variance, a log transformation was applied to all ELISA measurements. Tukey HSD heatmaps were generated to graphically show which testing conditions provided the best statistical power for detecting pairwise differences between material types and for examining variation between donors, heparin levels, models, and exposure time. The Tukey heatmap is useful when some of the pairwise differences have a known correct answer (e.g., different or not different) based on first principles or on previous experimentation. With the caveat that statistically significant differences in means may not necessarily indicate clinically meaningful differences in device/material performance, Tables 5a–d were developed as a straightforward means for ranking test material/device thrombogenicity based upon the Tukey Kramer HSD test and comparisons to positive control, negative control, and LMCD results. This ranking was also intended to roughly correspond to Table 3 NAVI scores. All data analyzed in this study used numbers directly obtained from test instruments and corrected only for the dilution factor used in the ELISA assay. None of the data received prior mathematical normalization, for example by subtraction of blank values, correction for blood dilution, conversion to percentages for example, of starting, final, or other values, or transformation of concentration to total amounts of measured factors. An exception here was transformation of Case Study 1 and 2 platelet count data on glass, PE, and No Material (negative) samples into % of the average No Material control. This data transformation was performed to examine the consistency of the glass and PE responses relative to assay validity conditions described in Reference 6. Lastly, on occasion a test tube or loop was lost due to a blood draw mishap or an air leak. In addition, in one study a set of samples from a repeated run was lost due to a cold storage mishap that lead to sample thawing.

**TABLE 5:** Simple scoring scheme that ranks test material/device in vitro thrombogenicity in terms of the various markers (A: TAT, B: F1.2, C: βTG, and D: Platelet concentration, [Plt]) relative to the positive control (glass), negative control (No Material/no material present), and the legally marketed comparator device (LMCD), for the particular medical device application. Results are determined using means comparisons from Tukey Kramer HSD tests on the various markers and within each group for example, heparin level, model, time, and so forth. The red lines mark the general threshold (from a regulatory perspective) of satisfactory versus nonsatisfactory scores (i.e., low concern vs. moderate to high concern)

A			
Thrombogenicity results (TAT)	TAT score	Comment	Thrombogenicity interpretation
TAT_Test_ < TAT_No Material_	–1	Test value is statistically significantly less than No Material	Antithrombogenic
TAT_Test_ ≈ TAT_No Material_	0	Test value is practically identical to No Material	Nonthrombogenic
TAT_No Material_ ≤ TAT_Test_ ≤ TAT_LMCD_	1	Test value clearly falls between No Material and LMCD	Low thrombogenicity
TAT_Test_ ≥ TAT_LMCD_	2	Test value is higher than LMCD, but not statistically	Predicate‐consistent thrombogenicity
TAT_LMCD_ < TAT_Test_ < TAT_Glass_	3	Test value is statistically higher than LMCD and less than glass	Moderate thrombogenicity
TAT_Test_ ≥ TAT_Glass_	4	Test value is greater than or equal to glass	High thrombogenicity

B			
Thrombogenicity results (F1.2)	F1.2 score	Comment	Thrombogenicity interpretation
F1.2_Test_ < F1.2_No Material_	–1	Test value is statistically significantly less than No Material	Antithrombogenic
F1.2_Test_ ≈ F1.2_No Material_	0	Test value is practically identical to No Material	Nonthrombogenic
F1.2_No Material_ ≤ F1.2_Test_ ≤ F1.2_LMCD_	1	Test value clearly falls between No Material and LMCD	Low thrombogenicity
F1.2_Test_ ≥ F1.2_LMCD_	2	Test value is higher than LMCD, but not statistically	Predicate‐consistent thrombogenicity
F1.2_LMCD_ < F1.2_Test_ < F1.2_Glass_	3	Test value is statistically higher than LMCD and less than Glass	Moderate thrombogenicity
F1.2_Test_ ≥ F1.2_Glass_	4	Test value is greater than or equal to Glass	High thrombogenicity

C			
Thrombogenicity results (βTG)	βTG score	Comment	Thrombogenicity interpretation
βTG_Test_ < βTG_No Material_	–1	Test value is statistically significantly less than No Material	Antiplatelet activating
βTG_Test_ ≈ βTG_No Material_	0	Test value is practically identical to No Material	Nonplatelet activating
βTG_No Material_ ≤ βTG _Test_ ≤ βTG_LMCD_	1	Test value clearly falls between No Material and LMCD	Low‐platelet activating
βTG_Test_ ≥ βTG_LMCD_	2	Test value is higher than LMCD, but not statistically	Predicate‐consistent platelet activating
βTG_LMCD_ < βTG_Test_ < βTG_Glass_	3	Test value is statistically higher than LMCD and less than Glass	Moderate platelet activating
βTG_Test_ ≥ βTG_Glass_	4	Test value is greater than or equal to Glass	High‐platelet activating

D			
Thrombogenicity results (platelets, [Plt])	Plt score	Comment	Thrombogenicity interpretation
[Plt]_Test_ > [Plt]_No Material_	–1	Test value is statistically significantly less than No Material	Antiplatelet consumptive
[Plt]_Test_ ≈ [Plt]_No Material_	0	Test value is practically identical to No Material	Nonplatelet consumptive
[Plt]_No Material_ ≥ [Plt]_Test_ ≥ [Plt]_LMCD_	1	Test value clearly falls between No Material and LMCD	Low‐platelet consumptive
[Plt]_Test_ ≤ [Plt]_LMCD_	2	Test value is lower than LMCD, but not statistically	Predicate‐consistent platelet consumptive
[Plt]_LMCD_ > [Plt]_Test_ > [Plt]_Glass_	3	Test value is statistically lower than LMCD and higher than Glass	Moderate platelet consumptive
[Plt]_Test_ ≤ [Plt]_Glass_	4	Test value is less than or equal to Glass	High‐platelet consumptive

Abbreviation: TAT, thrombin‐antithrombin complex; F1.2, prothrombin fragment F1.2; βTG, β‐thromboglobulin; Plt, platelet

## 3 RESULTS

### 3.1 High‐level observations from in vitro MTESs MTES1, MTES2, and MTES3

The impact of each study variable (material [M], model [Mo], time [T], blood donor [D], and heparin anticoagulation [H]) on each thrombogenicity measurement (TAT, F1.2, and βTG) is summarized in Table 6. The M, H, and D variables were common in all three studies, where M and H were highly significant across each measurement in each study (*p* < .0001), with the single exception (βTG and MTES3). Donor (D) showed mixed significance across the measurements and studies. The impact of T on thrombogenicity response was only addressed in MTES1 and was found to be highly significant (*p* < .0001) across each measurement. Mo was addressed in both MTES2 and MTES3 and found to be highly significant in each study and on each measurement with one exception (TAT in MTES2). The output of each MTES was further analyzed by graphical presentation and Tukey–Kramer‐based heat map analysis for the conditions of the study to predict the hypothetical material thrombogenicity order: Glass > (PE, Pebax, PEU) > No Material, and PEU ≥ PEU+H, across the three thrombogenicity measurements (where No Material is the tube or loop model without any test or control material).

**TABLE 6:** Summary of significant main effects and interactions from material thrombogenicity exploratory studies 1, 2, and 3 (MTES1, MTES2, and MTES3) for coagulation cascade activation (TAT and F1.2) and platelet activation (βTG). ● = variable not tested; NS = not significant (*p* > .05)

Variable ↓	Thrombogenicity measurement								
	TAT			F1.2			βTG		
MTES→	1	2	3	1	2	3	1	2	3
Material (M)	<0.0001	<0.0001	<0.0001	<0.0001	<0.0001	<0.0001	<0.0001	<0.0001	<0.0001
Heparin (H)	<0.0001	<0.0001	<0.0001	<0.0001	<0.0001	<0.0001	<0.0001	0.0349	NS
Donor (D)	<0.0001	NS	NS	<0.0001	NS	NS	NS	NS	0.0002
Time (T)	<0.0001	●	●	<0.0001	●	●	<0.0001	●	●
Model (Mo)	●	NS	<0.0001	●	0.0071	<0.0001	●	<0.0001	<0.0001
Significant interactions (*p* < .05)	MT MD MH DT DTH	MT MD MH DT	None	MH DT	MMo HMo DH	None	MT	MMo DH	MMo

Abbreviations: TAT, thrombin‐antithrombin complex; F1.2, prothrombin fragment F1.2; βTG, β‐thromboglobulin.

### 3.2 MTES1

This study examined responses in blood to the test materials over time using the test tube model (SDF blood filling) with nutation mixing, blood from three donors, exposure ratio = 9.0 cm^2^/ml whole blood, and heparin anticoagulation at two levels (0.6 and 1.0 U/ml). Results across all test variables are shown graphically in the Appendix. Generally, TAT, F1.2 and βTG measurements rise over the first 30 min (30–60 minutes) and show less increase over the subsequent 30 min intervals (60–90 min). In addition, measurement concentrations are clearly impacted by heparin level and the material present (Ps < 0.0001) and to some extent Donor (*p* <0 .0001 for TAT and F1.2; *p* > 0 .05 for βTG). Graphical and heat map analyses (see Appendix) illustrate the variability in predicting the material responses when data is combined across all three donors. The data also shows a significant difference between positive and negative controls across all 18 conditions (three timepoints x three blood donors x two heparin levels) and across all 54 measurements (18 conditions x three measurements [TAT, F1.2, βTG]). In addition, focusing on responses by each donor and at each heparin level showed the projected relationship Glass > (PE, Pebax, PEU) > No Material, and PEU ≥ PEU+H to be generally true for the thrombin activation markers (TAT and F1.2) but less so for the βTG platelet activation marker. The heat maps associated with 60 and 90 mins under low‐heparin show the strongest significance, with F1.2 data showing complete agreement. Under the high level of anticoagulant, the impact of immobilized heparin by the PEU+H material appears masked (2/6 TAT and F1.2 measurements) compared to testing under low‐heparin anticoagulation, where the TAT and F1.2 markers consistently captured the heparin bioactivity (~6/6 measurements).

### 3.3 MTES2 and MTES3

**FIGURE 4:**
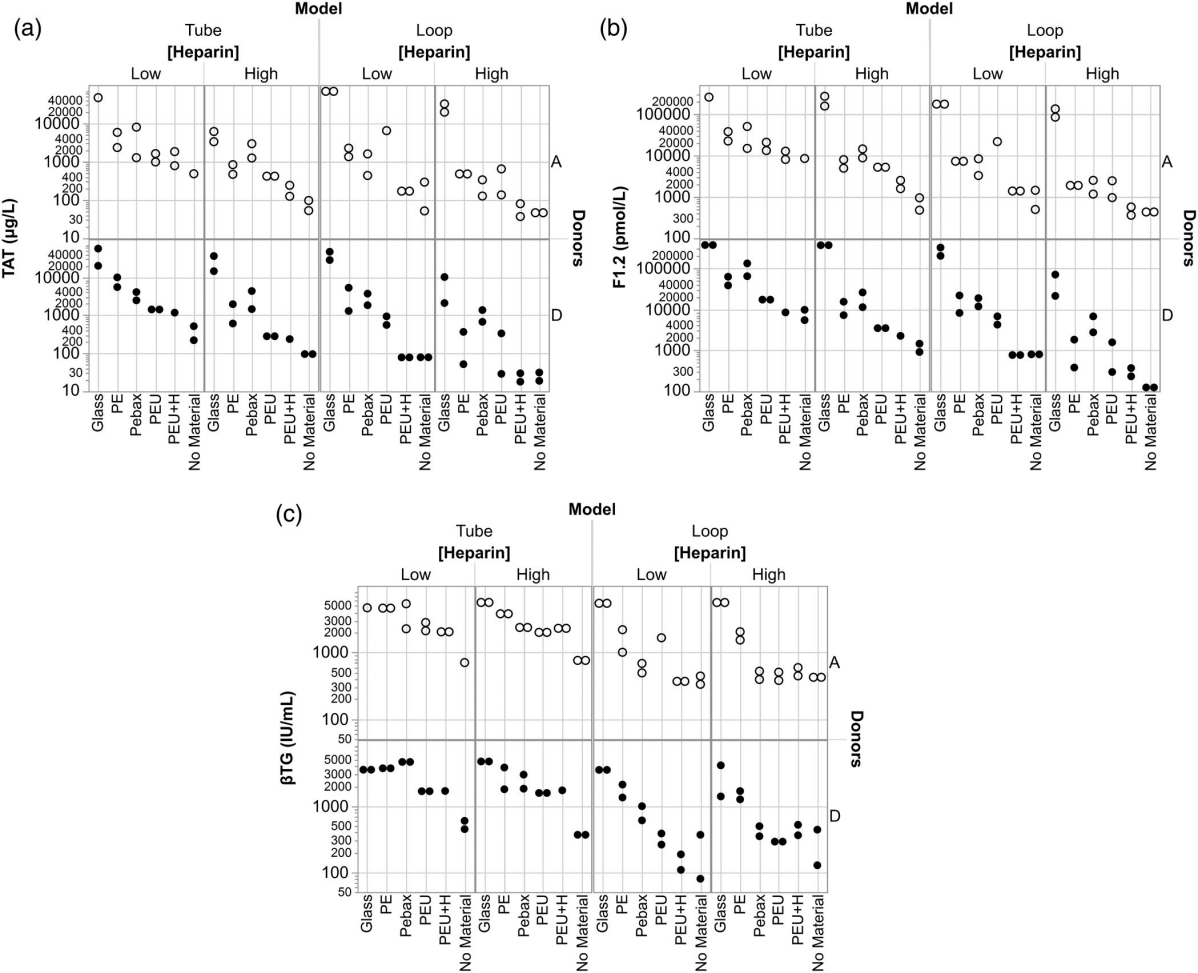
In vitro material thrombogenicity results for MTES3 showing (a) TAT, (b), F1.2, and (c), βTG responses to test materials across the variables Model, Heparin level, and Donor (A and D). Models: Tube model using VF blood filling with rocker‐roller mixing and loop model using pulsatile flow. Exposure ratio: 6.0 cm^2^/ml whole blood. PE, polyethylene; Pebax, polyether block amide; PEU, polyurethane; PEU+H, polyurethane with a heparin coating; No Material, Tube or Loop model without any test or control materials present. ANOVA showed the Donor factor to be significant only in the βTG measurements. ◯ = Donor A data; ● = Donor D data. (Heparin): Low = 1.0 U/ml; High = 2.0 U/ml. TAT, thrombin‐antithrombin complex; F1.2, prothrombin fragment F1.2; βTG, β‐thromboglobulin

These two exploratory studies had the same experimental design, and each examined the responses in blood to test materials using: two different models (tube model [VF] with rocker‐roller mixing vs. loop model [SDF] with pulsatile flow), 60‐min exposure duration, blood from two donors, and heparin anticoagulation at two levels (1.0 and 2.0 U/ml). The fundamental difference between the two studies was in the donors and exposure ratio used (donors A&B vs. A&D; and 9.0 vs. 6.0 cm^2^/ml whole blood, respectively). Results across all test variables are shown graphically for MTES2 (see Appendix) and MTES3 (Figure 4) and in heat map analysis for MTES2 (see Appendix) and MTES3 (Table 7). As would be expected, there was a general tendency for TAT, F1.2, and βTG responses to be lower with less material exposed to blood in MTES3 than in MTES2. A significant difference between positive and negative controls was observed in 23/24 conditions in MTES2 and 24/24 conditions in MTES3 (two models x two blood donors x two heparin levels x three measurements [TAT, F1.2, βTG]). In addition, comparison of the spread of measured responses across the materials in each study suggests a trend for more clear resolution of material differences at the lower exposure ratio (6.0 cm^2^/ml in MTES3), under the lower anticoagulation level (1.0 U/ml heparin), and in the loop model. As in MTES1, with focus on responses by each donor and at each heparin level MTES2 and MTES3 show that the projected relationship Glass > (PE, Pebax, PEU) > No Material, and PEU ≥ PEU+H was generally true for the thrombin activation markers (TAT and F1.2) but less so with the βTG platelet activation marker. Here too, ANOVA‐analysis (Table 6) indicates the test Material and Heparin factors had highly significant impact on the measurements (*p* < .0001), apart from the heparin impact on platelet activation (βTG *p* < .0349 in MTES2; *p* > .05 in MTES3). The Model factor also clearly had a strong impact on both coagulation and platelet responses (all *p* < .0001, with one exception: TAT in MTES2). Of the main effects, the D factor was not seen to greatly influence results in these two exploratory studies. The MTES3 heat map clearly shows better capture of the projected material thrombogenicity relationships compared to MTES2. The MTES2 heat map also illustrates the difference between the two models in capturing the projected materials responses under these conditions. With the greater levels of heparin anticoagulation in these two exploratory studies (1.0 and 2.0 U/ml) vs. MTES1 (0.6 and 1.0 U/ml), the impact of immobilized heparin on blood responses to the PEU+H material was clearly less observed, with detection limited to the loop model.

**TABLE 7:** Heat map of exploratory study MTES3. Red indicates a statistically significant difference as determined from Tukey–Kramer HSD analysis of data‐across the two models and with data from both donors. Comparisons with marginal statistical significance (.05 < *p* ≤ .07) are shown

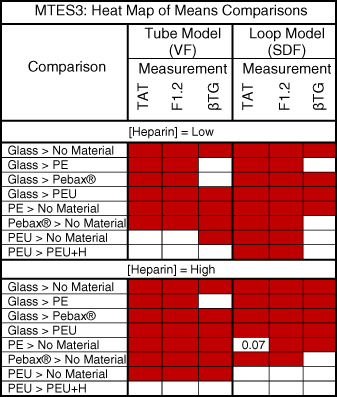						

Abbreviations: H, heparin; MTES, material thrombogenicity exploratory study; PE, polyethylene; Pebax, polyether block amide; PEU, polyurethane;PEU+H, polyurethane with a heparin coating; No Mat = No Material, no test or control material present; SDF, saline displacement filling; VF, vaccum fill; TAT, thrombin‐antithrombin complex; F1.2, prothrombin fragment F1.2; βTG, β‐thromboglobulin.

### 3.4 In vitro thrombogenicity results on devices or device materials tested under a select set of test variables and measurements

**FIGURE 5:**
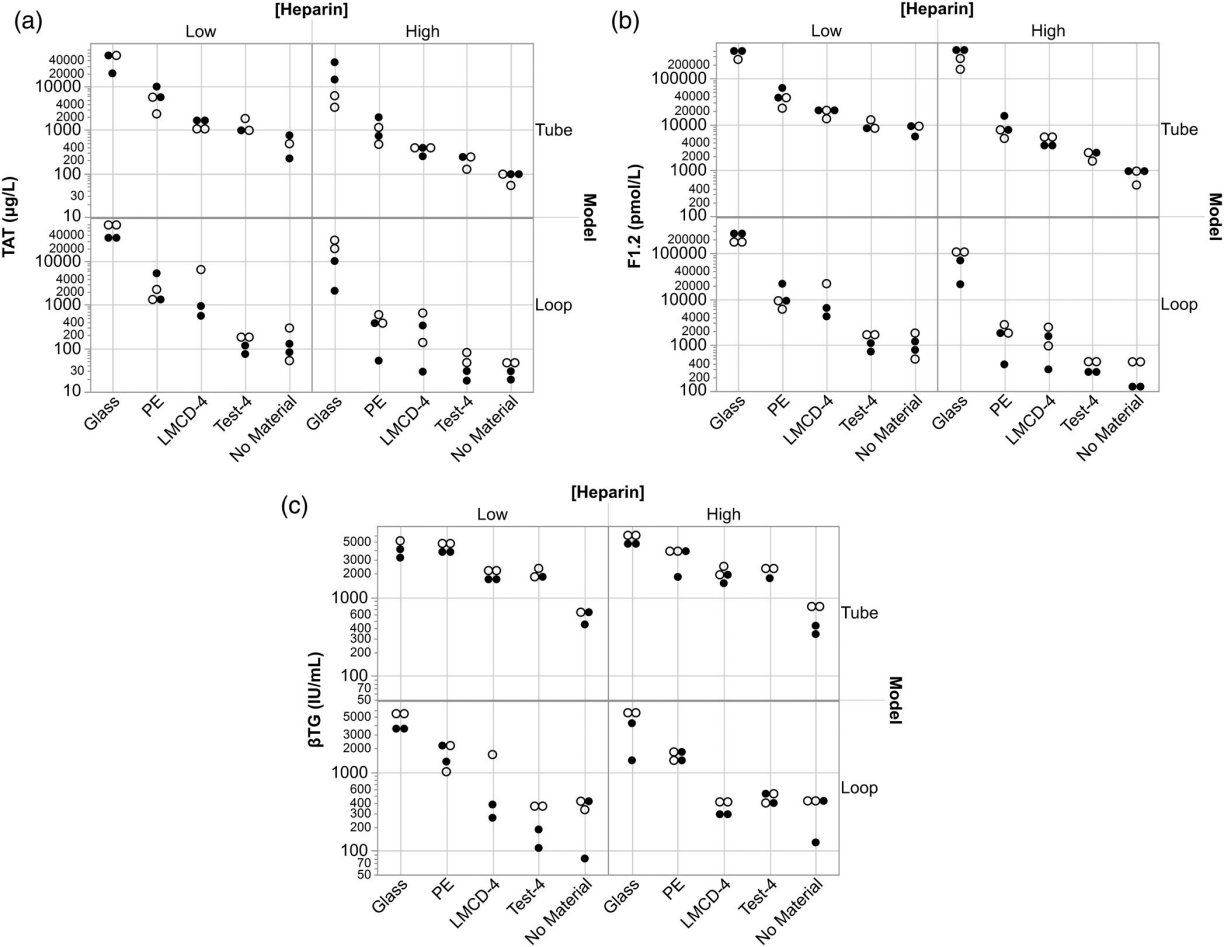
In vitro material thrombogenicity results for Case Study 4 showing (a), TAT, (b), F1.2, and (c), βTG responses in both Tube and Loop models to the test and various control materials. Scoring according to Table 5 reveals Test Material 4 (Test‐4) to be: 0–1 (TAT), 0–1 (F1.2), and 1–2 (βTG). LMCD, legally marketed comparator device/material; PE, polyethylene; No Material, Tube or Loop model without any materials present. ◯ = Donor A data; ● = Donor D data. [Heparin]: Low = 1.0 U/ml; High = 2.0 U/ml. TAT, thrombin‐antithrombin complex; F1.2, prothrombin fragment F1.2; βTG, β‐thromboglobulin

**FIGURE 6:**
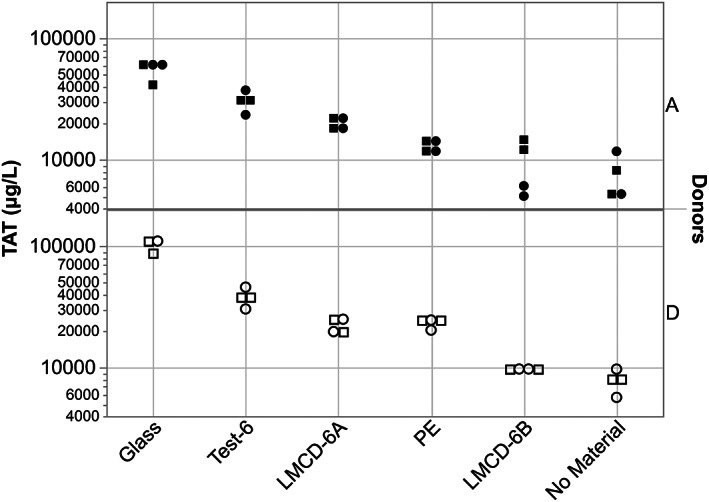
In vitro material thrombogenicity results for Case Study 6 showing the TAT responses to the test and various control materials. Scoring according to Table 5 reveals Test Device 6 (Test‐6) to be of moderate thrombogenicity concern, having TAT scores of 3 in blood from both donors. LMCD, legally marketed comparator device/material (two were used in this study–LMCD‐6A and LMCD‐6B); PE, polyethylene; No Material, tube model without any materials present. ● = Donor A Run 1, **■** = Donor A Run 2, ◯ = Donor D Run 1, □ = Donor D Run 2. Heparin anticoagulation was set at [Heparin] = 0.6 U/ml

**FIGURE 7:**
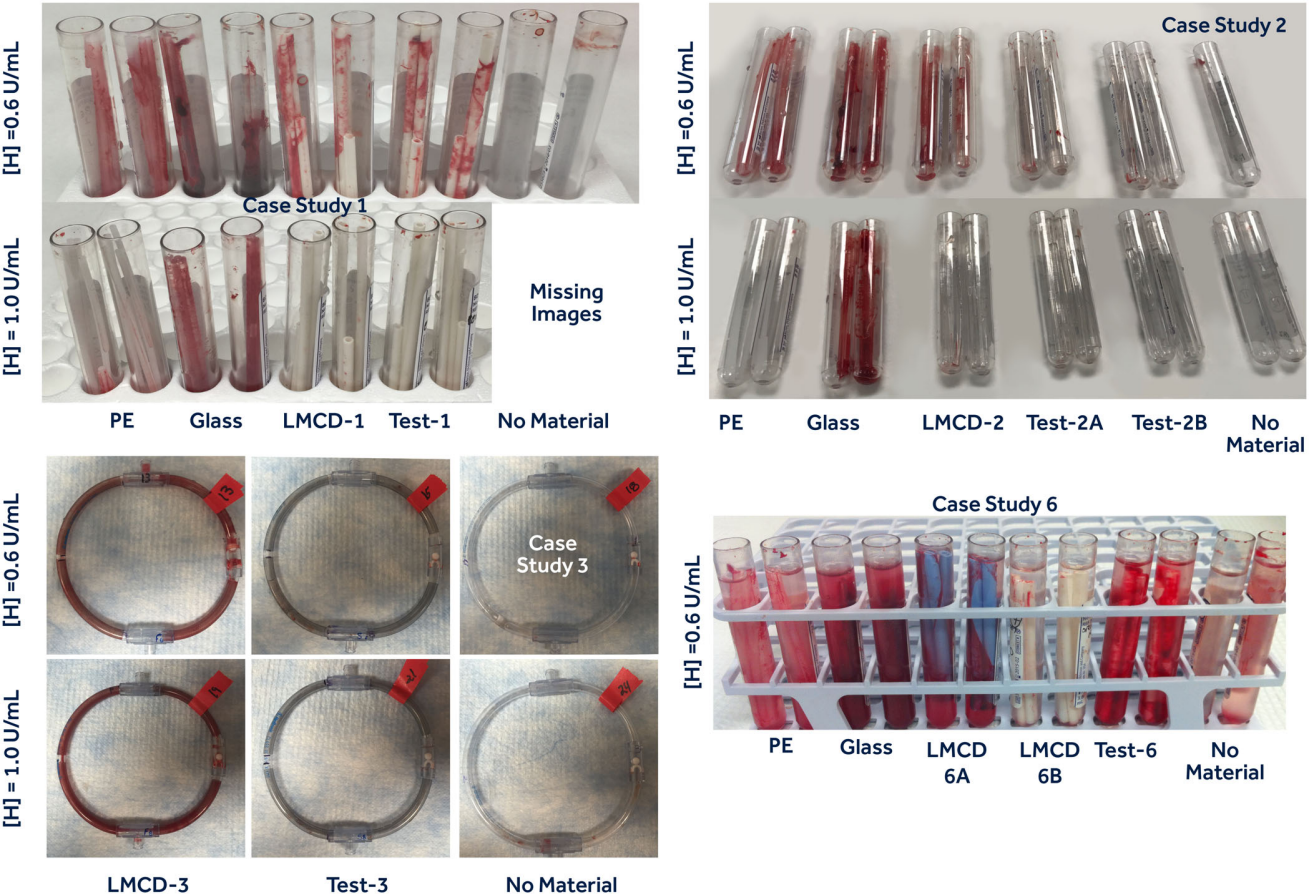
Representative images from devices/materials examined in in vitro Case Studies 1, 2, 3, and 6. LMCD, legally marketed comparator device; PE, polyethylene; No Material, no test or control material present

The results for the coagulation and platelet activation thrombogenicity measurements (TAT and/or F1.2; βTG and/or platelet loss) for each of the six in vitro case studies are shown graphically and by heat map tables. The results were scored based on the criteria described in Table 5, to identify results with classifications of low, moderate, and high concern. For illustrative purpose, Case 4 shows a material of low‐thrombogenicity concern (see Figure 5 and heat map Table 8) and Case 6 shows a test material of moderate thrombogenicity concern (see Figure 6 and heat map Table 9). The figures and heat map tables for the other cases are presented in the Appendix. All scores are summarized in Table 10. The test devices/materials in Cases 1 through 4 consistently revealed thrombogenicity measurements below the 2.0 threshold of concern. The test devices/materials in Case studies 5 and 6 revealed a number of conditions that gave rise to thrombogenicity scores above the 2.0 threshold of concern. Figure 7 shows representative gross images of some of the in vitro study samples after gentle rinsing with buffer to remove nonadherent blood.

**TABLE 8:** Heat map of comparisons of interest in in vitro thrombogenicity Case Study 4. Red indicates a statistically significant difference as determined from Tukey–Kramer HSD analysis of data‐across the two models and with data from both donors. Comparisons with marginal statistical significance (.05 < *p* ≤ .07) are shown

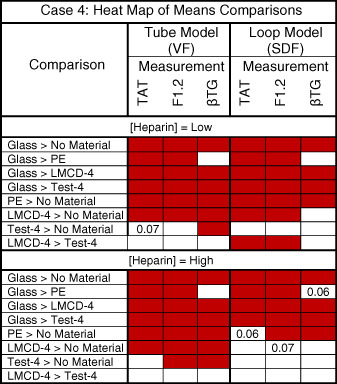						

Abbreviations: LMCD, legally marketed comparator device; MTES, material thrombogenicity exploratory study; PE, polyethylene; SDF, saline displacement filling; VF, vaccum fill; TAT, thrombin‐antithrombin complex; F1.2, prothrombin fragment F1.2; βTG, β‐thromboglobulin.

**TABLE 9:** Heat map of comparisons of interest in in vitro thrombogenicity Case Study 6. Red indicates a statistically significant difference as determined from Tukey–Kramer HSD analysis of data‐across the two models and with data from both donors. Comparisons with marginal statistical significance (0.05 < *p* ≤ 0.08) are shown

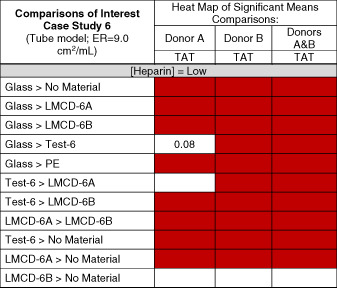			

Abbreviations: LMCD, legally marketed comparator device; PE, polyethylene; TAT, thrombin‐antithrombin complex.

**TABLE 10:** Applied in vitro thrombogenicity case studies on medical devices and medical device materials. Scoring was done according to Table 5. H = heparin, T = tube model, L = loop model. Two donors from a pool of five (A, B, C, D, and E) were used in each case study. Exposure ratio (ER) and duration was 6.0 cm^2^/ml blood and 60 min, except for Case 3, where the ER was four devices per loop. Heparin is in U/ml of whole blood

In vitro case study score summary										
Case study	Device/material	Model	Heparin	Donors	TAT score (score) = F1.2		βTG score		Platelet score	
					Low H	High H	Low H	High H	Low H	High H
1	Catheter X + Si oil	Tube	0.6 1.0	CD	1–2	1	1	1	1	0–1
2	Catheter Y + Endexo	Tube	0.6 1.0	AD	0–1	0–1	1	1	1	1
3	Pipeline + shield	Loop	0.6 1.0	DE	0–1	0	1	0–1	0	0
4	Catheter Z + H coating	Loop Tube	1.0 2.0	AD	T: 1(1) L: 0(0)	T: 1(1) L: 1(1)	T: 1 L: 1	T: 2 L: 1	NA	
5	Catheter W	Loop Tube	1.0 2.0	AD	T: 2(3) L: 1(2)	T: 3(3) L: 2(2)	T: 4 L: 2	T: 2 L: 1	NA	
6	Catheter X (new)	Tube	0.6	AD	3	NA	NA		NA	

Abbreviation: TAT, thrombin‐antithrombin complex; F1.2, prothrombin fragment F1.2; βTG, β‐thromboglobulin.

### 3.5 In vivo thrombogenicity results on the medical devices or medical device material

**FIGURE 8:**
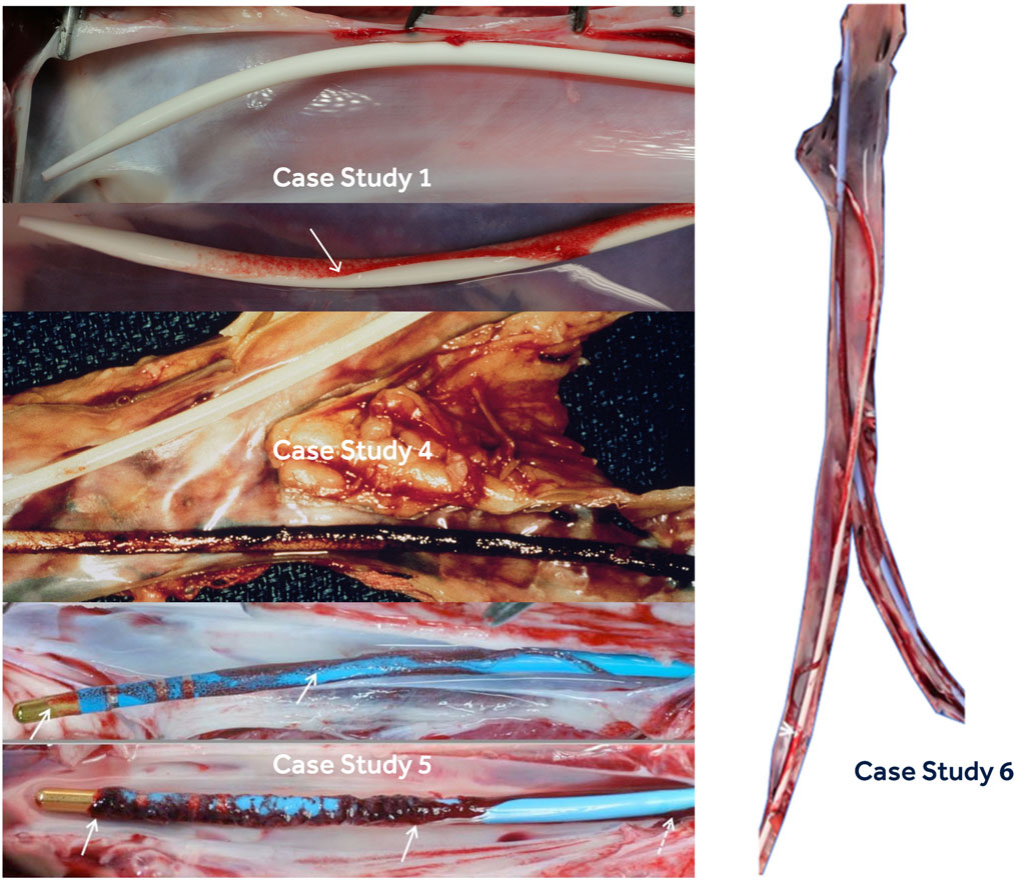
Representative explant images of some of the devices examined in the in vivo studies. Case Study 1 shows images of two Test Devices. Case Study 4 shows the Test Device (white catheter without thrombus) alongside the LMCD. Case Study 5 shows images of two Test Devices. Case Study 6 shows the LMCD alongside the Test Device (white catheter). NAVI scores for in vivo Case Studies 2 and 3 were estimated from References 16 and 18, respectively. The latter references should be consulted for in vivo images of these devices. LMCD, legally marketed comparator device; NAVI, nonanticoagulated venous implant

The in vivo thrombogenicity scores on the same or similar materials/devices evaluated in the in vitro thrombogenicity case studies are shown in Table 11. The test devices/materials in Cases 1 through 4 revealed passing (< 2.0) NAVI scores in the range of 0–0.7. The test devices/materials in Case studies 5 and 6 revealed nonpassing (> 2.0) NAVI scores in the range 2.5–3.5. For reference, Figure 8 shows representative explant images from the in vivo studies.

**TABLE 11:** In vivo NAVI (nonanticoagulated venous implant) studies wherein the highlighted test devices are the same as in the in vitro thrombogenicity Case Studies shown in Tables 2 and 11

In vivo case study	Device/material information		NAVI model info			NAVI score
	Descriptor^a^	Device/material	Model	Vein	n	
1	LMCD1	Catheter A	Canine	Femoral and jugular	3	1.0
	Test device 1	Catheter A + Si oil				0.7
2	LMCD2	Catheter B	Ovine	Jugular	12	0
	Test device 2	Catheter B + Endexo^b^				0
3	LMCD3	Pipeline stent	Laprine	Subclavian^c^	3	2
	Test device 3	Pipeline + shield				0
4	LMCD4	Catheter C	Canine	Femoral	1	4
	Test device 4	Catheter C + heparin coating				0
5	LCMD5	Catheter C	Canine	Femoral	4	2.4
	Test device 5	Catheter D				3.1
6	LMCD 6A and 6B	Dilator E (original) and F (other)	Canine	Femoral/IVC	2	2.25/0.25
	Test device 6	New dilator G				2.5/3.5

^a^ LMCD = legally marketed comparator device.

^b^ See in Reference 16.

^c^ See in Reference 18 (NAAI model).

## 4 DISCUSSION

Clinicians today have access to a multitude of life‐improving and life‐saving medical devices that involve contact with a patient's blood and each device presents its own inherent risk for triggering some degree of thrombosis. Here, both device‐specific and application‐specific factors come into play, such as: device material, geometry, surface area, and surface chemistry; and contact duration, fluid dynamics, vascular location, and supplementary anticoagulation and antiplatelet therapy. These important use condition factors can vary tremendously between applications and can impact the severity and risk of thrombosis. To establish safety and sound performance of these devices in blood, device manufacturers and regulatory agencies need to carefully consider the specific use conditions variables in each safety/risk assessment.^7^

Unfortunately, fundamental studies in hemocompatibility testing are not common, and existing studies often lack inclusion of appropriate controls (positive and negative) and consideration of basic use condition variables, such as impact of donor/patient variability and clinical anticoagulation (type and amount). This makes comparisons of data between laboratories difficult to interpret.^4^ Moreover, with so many different blood‐contacting devices, diversity in device clinical use conditions, countless test models, and limited in vitro‐to*‐*in vivo comparison studies, development of solid principles behind testing and creating blood compatible materials have arguably been hindered.^19^, ^20^

Given the crowd of factors that play a role in blood‐material/device interactions, a single model for screening devices/materials for thrombogenicity is not practical. In this study, we explored this limitation by using two fundamental blood‐contacting test models—one involving gentle mixing and the other flow‐based—while incorporating device and use conditions variables in the tests. The gentle‐mixing model utilized a simple test tube and several rocker‐roller mixers and blood draw techniques to impart a controlled immediate‐contact exposure of test and control materials with blood. This model approximates vascular environments where a device is exposed to low flow and periodic stasis of the blood, such as in lower extremity venous vasculature. The flow‐based model utilized small diameter closed loops as “circular test tubes” to likewise impart immediate test and control material blood contact, but under pulsatile flow conditions. The closed‐loop model logically lends itself to testing cylindrical‐geometry devices such as coronary and neurovascular stents, CPB tubing, and vascular grafts^8^, ^10^, ^11^, ^12^, ^21^ assuming perfect vessel (tubing) wall apposition. In contrast, devices of other geometries such as medium‐to‐large diameter catheters inserted into loops may alter or block flow, limiting device application in the small loop model. While making such models bigger has shown modest success,^22^, ^23^, ^24^ we chose to restrict our studies to these two models given the use condition simulation, the advantage of small volume, ease of use with fresh human blood, and the many practical drawbacks of large‐volume animal‐blood models. Unmistakably, the combination of small volume models with advanced multifactor experimental designs allowed a more robust study of the factors influencing blood‐material interaction.

### 4.1 Effect of the test model

The Model factor, examined in MTES2 and MTES3, was found to be significant across all thrombogenicity measurements, with only one exception‐TAT in MTES2 (Table 6). However, while material‐specific βTG, TAT, and F1.2 levels differ somewhat in each model, the relative material trends within each model generally remained similar. Still, examination of the MTES2 heat map (see Appendix) shows the Loop model to be a better predictor of the projected material thrombogenicity (Glass > [PE, PEU, Pebax] > No Material; PEU > PEU+H) compared to the Tube model, with predictivity best under low‐heparin anticoagulation. This model‐specific trend is less apparent in MTES3 heat map (Table 7) where both models show fair predictive agreement, with again the Loop model at low‐heparin appearing best able to detect difference in coagulation factors associated with the heparin coated test material. The main difference between MTES2 and MTES3 was the exposure ratio of 9.0 versus 6.0 cm^2^/ml, respectively. The less dense material packing and resultant more even blood mixing in MTES3 may have been an influential factor. It is noteworthy that the exposure ratio of 6.0 cm^2^/ml was arbitrarily chosen yet is recommended as a reasonable target in other types of studies.^25^ It is also a reasonable worse‐case exposure ratio as it is representative of the high‐exposure ratio seen in common extra corporeal membrane oxygenation (ECMO) procedures. In the latter case, an ECMO blood oxygenator with a surface area of 25,000 cm^2^ may come into contact with an average human adult blood volume of 5,000 ml, to create an exposure ratio of 5 cm^2^/ml. Some methods suggest blood interaction studies use higher exposure ratios (as high as 12 cm^2^/ml,^6^) to increase measurement signal‐to‐noise ratio. However, such high‐ratios depart greatly from typical clinical use conditions. Moreover, at such high‐exposure ratios the important ability of blood to evenly distribute and mix over the material surface can be substantially diminished, as we observed at even 9 cm^2^/ml in MTES2.

### 4.2 Effect of device use conditions variables

As each medical device application involves diverse patients, unique device blood‐contact durations, varying anticoagulation regimens, and devices composed of various materials, we chose to examine donor (D), blood exposure time (T), heparin anticoagulation (H), and material (M) as main variables in our exploratory studies. Table 6 summarizes the significant main effects and interactions observed in these material thrombogenicity exploratory studies (MTES 1–3). The Material factor was found to be highly significant (*p* < .0001) across βTG, TAT, and F1.2 measurements in all three studies. This was driven across all study conditions in part by the significant difference in responses between positive the Glass (positive) and No Material (negative) controls. The purely polymeric test materials (PE, PEU, and Pebax) generally showed intermediate levels of coagulation and platelet activation. The heparin‐modified material PEU+H showed the anticipated trend for lower responses in blood than its non‐modified counterpart.

Heparin anticoagulation was found to be a highly influential variable across all MTES studies, revealing significance (*p* < .0001) in all measured factors except for βTG in MTES3 (*p* > .05). The level of heparin anticoagulation was carefully chosen in our studies since unpublished work showed levels less than 0.6 U/ml have a high risk of complete coagulation in all samples and that 0.6 U/ml has proven to be relatively safe to avoid excessive coagulation.^8^, ^11^, ^12^ Conversely, levels of heparin greater than 2.0 U/ml were anticipated to generate minimal/background responses across all conditions and materials due to the known therapeutic effectiveness of heparin to quench thrombotic processes at or above this level. In general, heparin levels of 0.6, 1.0, and 2.0 U/ml whole blood were sufficient to consistently avoid excessive coagulation yet still see significant differences between positive (Glass) and negative (No Material) controls. The 0.6 and 1.0 U/ml whole blood heparin concentrations used in MTES1 worked well to simulate a “challenging” (low) and “conventional” (moderate) level of anticoagulation, whereas the 1.0 and 2.0 U/ml levels as used in MTES 2 and MTES3 were clearly associated with diminished responses in blood. Based upon these findings, heparin levels greater than 2.0 U/ml in simple tube and loop models at exposure ratios of 6–9 cm^2^/ml whole blood are not likely to produce βTG, TAT and F1.2 responses that are statistically different across varying test and control materials. These in vitro observations are consistent with our in vivo observations, and those of others, when applying the NAVI and anticoagulated venous implant (AVI) models, which use no anticoagulation and full anticoagulation in in vivo testing of medical devices/materials for thrombogenicity.^3^, ^7^

Regarding the use of multiple donors in our exploratory studies, the Donor factor showed mixed significance across the βTG, TAT, F1.2 measurements. The understanding of differences in coagulation responses between subjects and within individual subjects over time account for this observation.^26^, ^27^, ^28^ Importantly, despite having a significant Donor effect in some of the MTES studies, the relative responses between the materials were similar in blood from different individuals. This suggests that such testing using only a small number of donors will generally give consistent results and a fair estimate of material thrombogenicity. It is important to note, however, that baseline coagulation marker levels in healthy donors (this study) may have less variability compared to those of a typical patient population, where elevated and varying levels of factors may be due to underlying disease state and comorbidities.

Only MTES1 examined blood exposure time as a variable. This revealed the expected trend of coagulation and platelet activation responses increasing with time and appearing to plateau (by 90 min under these conditions). Understanding the dependence on time of indicators of hemocompatibility is essential as many molecular reactions involved in coagulation follow a sigmoidal relationship comprised of initiation, propagation, and termination phases.^28^ In the initiation phase, detecting material‐specific differences in coagulation responses may be challenging due to measurement error at low levels of reaction. At the opposite extreme, differences in measured factors across test materials in the termination phase may be difficult to differentiate, as reactions may have gone to completion (maximum levels) given the finite amounts of profactors available. This tenet dictates that differences in material thrombogenicity are most detectable before reactions reach termination phase and while reactions are in the propagation phase.

### 4.3 Correlation of in vitro to in vivo thrombogenicity

The second half of this investigation examined the ability of simple multiparameter in vitro thrombogenicity tests to give results comparable to in vivo models. A common and somewhat controversial in vivo approach to material/device thrombogenicity assessment is the NAVI model (and its anticoagulated model counterpart‐AVI).^1^ A critical factor that guides interpretation of device/material thrombogenicity in these models is inclusion of a LMCD (a clinically approved device used in the same application). To assess device risk, such studies are evaluated according to the criteria in Table 3 and the test device/material is examined for score/response similarity to the LMCD. Table 11 summarizes the in vivo thrombogenicity scores on the same/similar materials used in the case studies. The in vivo results reveal the test materials/devices involved in case studies 1–4 gave passing NAVI scores while those involved in case studies 5 and 6 showed nonpassing scores.

Table 12 contrasts the in vivo and in vitro thrombogenicity test results on the six medical‐device/material case studies. The in vitro TAT/F1.2, βTG, and platelet count measurements across case studies 1–4 under high heparin produced 14/15 scores in the 0–1 category and 1/15 score in the category of 2. The same distribution of in vitro scoring was seen under low heparin in these cases. This confirmed the materials to be low−/nonthrombogenic and low−/nonplatelet activating under in vitro testing and Table 5 interpretation. The in vivo thrombogenicity scores in these same four cases were also low (0–0.7), showing solid agreement. Case studies 5 and 6, on the other hand, revealed 2/6 in vitro scores of moderate thrombogenicity under high heparin and 3/7 in vitro scores of moderate‐thrombogenicity to high‐platelet activation concern at low heparin. The in vivo thrombogenicity scores in these same two cases indicated nonpassing scores (3.1 in Case 5 and 2.5–3.5 in Case 6). Using the basis of any in vitro result (in either model) above 2.0 to indicate an overall nonpassing response (erring on the side of a false positive being safer than a false negative), seven out of eight in vitro*/*in vivo comparisons (88%) show reasonable agreement. Here, our in vitro approach based upon use condition (uc) blood exposure offers the advantage (and challenge) of material (M) thrombogenicity assessment based upon a battery of relevant molecular test results that is, protein indicators of thrombin (T) formation (TAT and F1.2 proteins) and platelet (P) activation (A) [βTG and platelet counts]. The numerical nature of these “ucMTPA” measurements also allow pass/fail assessment via routine statistical analyzes, rather than assessment via a single subjective score. Interestingly, comparison of gross images from in vitro case studies (Figure 7) to gross images from corresponding in vivo studies (Figure 8) reveals similarities in relative amounts of gross surface thrombus buildup on the materials at the low‐anticoagulation level. These in vitro methods suggest, albeit crude, a use condition “test tube NAVI” model. These findings indicate that bioanalytical measurement of molecular indicators of thrombosis, and material gross appearance after in vitro exposure to fresh human blood, can give sound estimates of material/device thrombogenicity consistent with in vivo thrombogenicity studies.

**TABLE 12:** Summary table showing level of agreement of in vivo and in vitro thrombogenicity test results. Note: comparison of in vitro scores under high heparin are excluded in this comparison, as the in vivo models were conducted at low/no heparin anticoagulation

In vitro score (tube [T] and or loop [L] model)												
Case	In vivo^a^ score	Model	TAT (F1.2)		βTG		Platelets		Agreement of in vitro and in vivo scores?			
			H = low	H‐high^c^	H = low	H‐high^c^	H = low	H‐high^c^	%^d^	Total	Y/N (yes/no)	Y/N
1	0.7	T	1	0–1	0–1	0–1	0–2	0–1	3/3	15 out of 15	Y	5/5
2	0	T	0–1	0–1	1	0–1	0–1	0–1	3/3	–	Y	–
3	0	L	0	0	0–1	0	0	0	3/3	–	Y	–
4	0	T	0–1 (0–1)	0–1 (0–1)	1	2	NA	NA	3/3	–	Y	–
–	–	L	0–1 (0–1)	0–1 (0–1)	0–1	0–1	–	–	3/3	–	Y	–
5	3.1	T	2 (3)	3 (3)	4	2	NA	NA	2/3	3 out of 7	Y (majority)	2/3
–	–	L	1 (1)	2 (2)	2	2	–	–	0/3	–	N	–
6	2.5, 3.5^b^	T	3	NA	NA	NA	NA	NA	1/1	–	Y	–
**–**									18/22		**–**	7/8
									(82%)			(88%)

^a^ All NAVI model results except Case 3 (see in Reference^17^).

^b^ Femoral and IVC model scores, respectively.

^c^ Comparison of NAVI scores to in vitro scores @ (H) = High not done.

^d^ TAT, F1.2, and βTG scores at (H) = Low.

### 4.4 Limitations

Given the exploratory nature of this work, there were some notable drawbacks. To start, blood exposure to air, the degree of blood dilution, blood mixing motion, the amount of material exposed to blood (exposure ratio), and the type of anticoagulant (including combinations of anticoagulants and antiplatelet drugs) all clearly have some degree of influence on blood responses. However, these experimental variables were not implemented systematically into our exploratory study designs, impeding thorough assessment of their impact. Also, the small number of healthy blood donors used in the exploratory studies (4) and in the case studies (5) is likely only moderately representative of individuals in the human population and not typical of the patient population that receives medical device therapies. From our studies and those of others^26^, ^27^ it is expected that differences in the human population and random selection of blood donors will lead to in vitro blood interaction studies conducted on blood that comes from a spectrum of individuals, for example, from low to high‐level responders. Nonetheless, treating blood donor as a variable in blood‐device/material interaction studies offers an understanding of blood interaction response variability with inter‐ and intra‐donor comparison of test material to LMCD, positive and negative controls. Conversely, blood interaction studies that use single source or pooled blood lose this information and can give a potential false sense of consistency of patient blood responses.

To better simulate use conditions, our studies utilized a unique blood draw procedure that involved blood drawn directly into the models and resultant immediate blood exposure to test and control materials. However, no comparison of this procedure was made to other more common approaches of test material exposure to blood, for example, approaches that involve a time delay between the blood draw and blood use, and the use blood with prior contact with one or more nontest/nonmodel materials. The importance of using fresh blood within 4 hr of blood collection (and preferably within 2 hr) has been reported.^29^ Interestingly, examination of our platelet count data expressed relative to the No Material control according to Reference 6 showed the HDPE reference biomaterial to be consistently outside (60–80%) the assay validation condition of 80–120% (see Appendix). This low level of HDPE platelet reactivity has been reported by others using alternative blood preparation/exposure methods.^30^, ^31^ This difference in HDPE reactivity, which is supported by extensive additional unpublished work in our lab using a commercially available HDPE reference biomaterial suggests that blood “freshness” and exposure conditions may influence thrombogenicity measurements. Conversely, exposure of test materials to aged blood, citrated blood bank blood, recalcified and heparinized blood, or fractionated blood (e.g., blood plasma, platelet rich plasma, fresh or pooled/frozen serum) deviates significantly from most use conditions and may give unreliable results.

On the material side, our screening study use of a heparin‐coated material that excluded heparin coating process optimization steps (that increase immobilized heparin bioactivity and uniformity) led to some observations of nonstatistically significant trends between coated and uncoated materials. Despite this drawback, the expected trend was apparent.

Regarding models, we chose to examine two well‐established simple in vitro models, yet each model presents its own drawbacks. One drawback of the Tube model is the type and degree of blood mixing and resultant interaction with test surfaces that occurs relative to the type of mixer used and the sample geometries. For example, some mixers rely on the presence of an air space for a sloshing mixing effect. Some consider this type of blood contact to be potentially too far removed from physiological conditions. For the Loop model, one disadvantage of these “circular test tubes” is the requirement of a small check valve to support pulsatile flow generation and a computer‐controlled microstepper motor to impart pulsatile flow through a controlled rotational pattern. Our simple and easily formed first generation ball‐and‐cage valve performs well but also may elicit some flow‐induced thrombogenicity in the baseline negative (No Material) control at low anticoagulation. In addition, test devices and materials that greatly obstruct the lumen can disturb the pulsatile flow, which may confound results if test material geometries and resultant obstruction differ greatly.

Finally, the general experimental design used in the in vitro case studies (Figure 5), calling for two donors, two heparin levels, and n = 4 (runs of n = 2 repeated twice), and measurement of one to three indicators of material thrombogenicity, has room for both simplification, and further expansion. For example, such simplification was applied in Case study 6, which utilized only one heparin level and involved only one measurement for thrombogenicity (TAT), and yielded solid results. Expansion of test measurements could be through inclusion of other recognized indicators of hemocompatibility, such as complement protein activation and hemolysis testing, and correlative studies with other potentially more sensitive tests, such as platelet activation assessment through whole blood flow cytometry of P‐selectin. In addition, while these studies were limited to use of heparin as the anticoagulant, studies that include antiplatelet drugs and thrombin inhibitors may help elucidate whether thrombin generation is predominantly by contact activation of the FXII pathway, or via amplification on the phosphatidylserine‐rich surface of activated platelets, or both. Moreover, with the amount of blood or plasma called for in our tests, it is envisioned that a multitude of factors can be assessed simultaneously in a single study. Clearly, additional studies that show meaningful results achieved under further simplified study designs, and correlative studies with other recognized indicators of material thrombogenicity, are warranted. Additional in vitro*/*in vivo comparisons studies to verify the findings in this report are also deserved.

## 5 SUMMARY

In the 19th century the famous physician Rudolf Virchow first alluded to thrombosis being influenced by a small number of critical factors.^32^ Clearly the mechanisms of thrombosis are much better understood today, and in association with cardiovascular devices thrombosis can be viewed to be influenced by at least six important factors: (1) disturbed/nonphysiological flow through/around devices (2) blood “hypercoagulability”, a term describing a perturbation in the mechanisms of hemostasis and/or differing coagulation potentials recognized to exist between and within individuals (3) vascular/endothelial injury, a highly influential factor that can impact thrombosis associated with medical devices in terms of tissue trauma due to the device or implant procedure, as well as in vitro blood studies in terms of trauma associated with the blood draw technique and general blood handling (4) anticoagulants, antiplatelet drugs, and antithrombotic therapies–powerful drugs that can remarkably impact thrombus formation associated with medical devices (5) medical device materials and the growing list of surface‐modified and drug‐eluting materials that impact molecular and cellular interactions at the blood‐contacting interface, and (6) general device application specifics, which addresses unique device use conditions such as blood‐contact duration and surface area of exposure, macroscopic and microscopic material geometry, tissue engineering factors for example, living endothelial cells applied to devices,^33^, ^34^, ^35^, ^36^ and target vasculature (venous vs. arterial, heart vs. brain, etc.). This report shares a first principles‐based approach to designing and executing studies that evaluate blood‐material interactions with respect to this hexad of important factors. We describe applying a use condition (uc) approach to thrombogenicity testing that uses modern bioanalytical tools to assess medical device materials (M) based upon their capacity to induce thrombin (T) and platelet (P) activation (A) (a “ucMTPA” test). Based on these studies, in vitro models using human blood with minimal trauma and immediate exposure to test materials within specified ranges of exposure ratio, exposure time, and heparin anticoagulation, gave bioanalytical measurements of coagulation and platelet activation that were consistent with acute in vivo device/material thrombogenicity assessments.

## Supporting information


**Appendix** S1: Supporting Information.

## Endnote

^1^ Note: the analogous models for arterial (A) devices are referred to as the nonanticoagulated arterial implant (NAAI) model and the anticoagulated arterial implant (AAI) model.^3^, ^7^
