# Supplementary material for: In vitro methodology for medical device material thrombogenicity assessments: A use condition and bioanalytical proof‐of‐concept approach
Source: J Biomed Mater Res B Appl Biomater. 2020 Sep 14;109(3):358–76. doi: 10.1002/jbm.b.34705 (PMC7821245; doi:10.1002/jbm.b.34705)
Supplement: Supplementary file 1 — Appendix S1: Supporting Information. [file JBM-109-358-s001.zip › JBMB_34705_Mike Wolf et al CHAPTER APPENDIX 782020.docx]

**APPENDIX**

**In vitro methodology for medical device material thrombogenicity assessments: a use condition and bioanalytical proof-of-concept approach**

*Blood Exposure Test Models*

*Test Tube Model.* The vacuum filling (VF) blood draw technique in this model was accomplished by re-establishing the appropriate vacuum in the test tube to draw in the desired volume of blood using standard hematology venipuncture practice. The target vacuum was determined using BD No Additive Vacutainer® tubes (reference no. 366703) containing the exact amounts of test (or control) material and anticoagulant, and creating a standard regression curve of vacuum vs. amount (mL) H_2_0 drawn. The vacuum was recreated in the tubes through connection of the sealed/capped tubes to a mechanical syringe pump (KDS200 Dual-Syringe Pump, KD Scientific) using BD blood collection sets, air withdraws of 5 to 20 mL, and leaving in place yet pinching closed the collection set line to maintain the new test tube vacuums. The resulting blood concentration was 94% due to the added 200 µL anticoagulant. The alternative saline displacement/no-air-exposure draw technique was accomplished by priming test- and control-material loaded capped tubes with 100% heparinized buffer. A 5-mL ‘withdraw-only’ safety syringe attached to an 18G x 3.5inch BD spinal needle (BD reference no. 405184) was inserted through the cap to the bottom of the tube. ‘Withdraw-only’ was achieved by adding a check valve (Qosina parts 11582, 12090, 17642, Ronkonkoma, NY) between the spinal needle and syringe that allowed only withdraw and prevented injection. With the tube inverted, a BD blood collection set (BD reference no. 367281) connected to the donor antecubital vein was inserted through the tube cap alongside the spinal needle. Withdraw of a specified volume of prime solution into the 5-mL withdraw-only syringe then draws in the same volume of donor blood without air exposure. The volume of residual saline remaining in the tube after blood draw fill was deliberately selected beforehand to establish the prime solution heparin concentration that gives the desired final blood heparin concentration and % blood dilution. Note: in using the SDF draw technique, blood in the tube will not be 100%. In these studies, the blood dilution was set to 65±2% (based upon 3.0 mL blood draw volume, test tube volume of 5.5 mL, and the volume of the test or control material).

*Dynamic Closed-loop Model*. Pulsatile blood flow within the closed loops was achieved by subjecting loops to a repeating rotational pattern using a computer-programmable rotary actuator pulsatile drive system (Figure A1). The precise motion profile involved a 0.5-1.0-0.5 acceleration-constant speed-deceleration (rev/s^2^-rev/s-rev/s^2^) pulse followed by an 800 millisecond pause. This motion imparts a pulsatile flow profile of 60 pulses per minute to the blood inside a No Material/PVC loop with peak and average flow rates of 300 and 100 mL/minute (Figure A2). This flow rate is representative of the lower end of blood flow estimated for small 3-4 mm ID arteries [9]. Each loop was constructed of one 16.5 cm and two 7.5 cm pieces of medical grade PVC tubing (3/16” x 1/32” ID x WT), one 2.5 cm piece of 1/4” x 1/16” medical grade PVC tubing, two injection/withdraw ports (84048 T-connector with 80149 Male Luer Lock Injection Site, Latex Rubber; Qosina, Edgewood, NY, USA), and one 3/16 diameter PTFE sphere and one half of a PTFE O-ring (7/32” ID x 1/16” CS) (Amazon.com, Inc). The PTFE parts and the short 2.5 cm PVC part were used to form a simple integral ball-and-cage check valve that allows easy opening and closure imparted by fluid momentum driven flow. All connector and PVC joints were solvent bonded using tetrahydrofuran and tested to be leak-free. The internal volume of the test loops was gravimetrically determined to be 6.3 mL. Before solvent bonding, loops were individually loaded with a specific surface area of control and test materials to give the target exposure ratio (6 or 9 cm^2^/mL whole blood). At the start of each experiment, loops were 100% filled/primed with a specific concentration of heparin in Plasma-Lyte™ (as in SDF tube model). A BD blood collection set connected to a donor antecubital vein was inserted into the injection/withdraw port (Port 1- see main paper Figure 2). A 10-mL withdraw-only syringe (see main paper) attached to the injection/withdraw Port 2 was used to withdraw 3.2 or 5.0 mL prime solution and concomitantly draw in 3.2 or 5.0 mL venous blood through Port 1 to achieve target 50% and 80% final blood concentrations, respectively. The blood-filled loops were placed on the pulsatile drive system for 30 to 90 minutes @ 37°C and 60 pulses per minute. As in the tube studies, loops were filled in randomized order, B_i_ and B_f_ samples were collected, and equivalent post blood exposure steps were applied to quench blood activation processes, to prepare blood CBC and plasma ELISA samples, and to record visible thrombus on the materials in the loops.

**List of Appendix Tables**

**Table A1.** Heat map of exploratory study MTES1. Red indicates a statistically-significant difference as determined from Tukey-Kramer HSD analysis of data across all three donors. Comparisons with marginal statistical significance (0.05 < P ≤ 0.07) are shown.

**Table A2.** Heat map of exploratory study MTES2. Red indicates a statistically-significant difference as determined from Tukey-Kramer HSD analysis of data across the two models and with data from both donors. Comparisons with marginal statistical significance (0.05 < P ≤ 0.07) are shown.

**Table A3.** Heat map of comparisons of interest in *in vitro* thrombogenicity Case Study 1. Red indicates a statistically-significant difference as determined from Tukey-Kramer HSD analysis of data across the two models and with data from both donors.

**Table A4.** Heat map of comparisons of interest in *in vitro* thrombogenicity Case Study 2. Red indicates a statistically-significant difference as determined from Tukey-Kramer HSD analysis of data across the two models and with data from both donors. Comparisons with marginal statistical significance (0.05 < P ≤ 0.07) are shown.

**Table A5.** Heat map of comparisons of interest in *in vitro* thrombogenicity Case Study 3. Red indicates a statistically-significant difference as determined from Tukey-Kramer HSD analysis of data across the two heparin levels and with data from both donors.

**Table A6.** Heat map of comparisons of interest in *in vitro* thrombogenicity Case Study 5. Red indicates a statistically-significant difference as determined from Tukey-Kramer HSD analysis of data across the two models and with data from both donors. Comparisons with marginal statistical significance (0.05 < P ≤ 0.07) are shown.

**List of Appendix Figures**

**Figure A1.** Pulsatile Drive System that applies a computer programmed motion profile to test loops friction fit onto the loop mounting drum.

**Figure A2.** Pulsatile wave form in closed loop model caused by a repeating 0.5-1.0-0.5 acceleration-constant speed-deceleration (rev/s^2^-rev/s-rev/s^2^) pulse followed by an 800 millisecond pause. This motion imparts a pulsatile flow (pulse rate of 60 min^-1^) profile to the fluid inside the loop with peak and average flow rates of approximately 300 and 100 mL/min.

**Figure A3.** *In vitro* material thrombogenicity results for MTES1 showing TAT (A), F1.2 (B), and βTG (C) responses to test materials across the variables Time, Donor (A, B, and C), and Heparin level. Model: tube using SDF blood filling with nutation mixing. Exposure ratio: 9.0 cm^2^/mL whole blood. PE = polyethylene, Pebax = polyether block amide, PEU = polyurethane, PEU+H = heparin coated polyurethane, and No Material = test tube without any materials present. **○** = heparin anticoagulation at 0.6 U/mL; ● = heparin anticoagulation at 1.0 U/mL.

**Figure A4.** *In vitro* material thrombogenicity results for MTES2 showing TAT (A), F1.2 (B), and βTG (C) responses to test materials across the variables Model, Heparin level, and Donor (A and B). Models: tube using VF blood filling with rocker-roller mixing and loop using pulsatile flow. Exposure ratio: 9.0 cm^2^/mL whole blood. PE = polyethylene, Pebax = polyether block amide, PEU = polyurethane, PEU+H = heparin coated polyurethane, and No Material = test tube/loop without any materials present. **○** = heparin anticoagulation at 1.0 U/mL (Low); ● = heparin anticoagulation at 2.0 U/mL (High).

**Figure A5.** *In vitro* material thrombogenicity results for Case Study 1 showing TAT (A), βTG (B) and Platelet (Plt; C) responses to Test Device 1 (Test-1) and various control materials. Scoring according to Table 5 reveals Test Material 1 (Test-1) to be: 1-2 (TAT), 1 (βTG), and 0-1 (platelets). LMCD = legally marketed comparator device/material; PE = polyethylene; No Material = test tube without any materials present. ● = Donor C Run 1, ■ = Donor C Run 2, **○** = Donor D Run 1, □ = Donor D Run 2. [Heparin]: Low = 0.6 U/mL; High = 1.0 U/mL

**Figure A6.** *In vitro* material thrombogenicity results for Case Study 2 showing TAT (A), βTG (B) and Platelet (Plt; C) responses to Test Materials 2A and 2B (Test-2A and Test-2B) and various control materials. Scoring according to Table 5 reveals that Test Materials 2A and 2B performed similarly, with scores of: 0-1 (TAT), 1 (βTG), and 0-1 (platelets). LMCD = legally marketed comparator device/material; PE = polyethylene; No Material = test tube without any materials present. ● = Donor A Run 1, ■ = Donor A Run 2, **○** = Donor D Run 1, □ = Donor D Run 2. [Heparin]: Low = 0.6 U/mL; High = 1.0 U/mL

**Figure A7.** *In vitro* material thrombogenicity results for Case Study 3 showing TAT (A), βTG (B) and Platelet (Plt; C) responses to the Test Device (Test-3) and control materials. Scoring according to Table 5 reveals Test Material 3 (Test-3) to be: 0-1 (TAT), 0-1 (βTG), and 0 (platelets). LMCD = legally marketed comparator device/material; No Material = test loop without any materials present. ● = Donor D, ■ = Donor E. [Heparin]: Low = 0.6 U/mL; High = 1.0 U/mL

TAT_No Material_

< TAT_Test-3_

< TAT_LMCD-3_

Score = 1

TAT_No Material_

≈ TAT_Test-3_

< TAT_LMCD-3_

Score = 0

**Figure A8.** *In vitro* material thrombogenicity results for Case Study 5 showing TAT (A), F1.2 (B) and βTG (C) responses in both Tube and Loop models to Test Material 5 (Test-5) and various control materials. Scoring according to Table 5 reveals Test Material 5 (Test-5) to be: 1-3 (TAT), 2-3 (F1.2), and 2-4 (βTG). LMCD = legally marketed comparator device/material; PE = polyethylene; No Material = test tube without any materials present. ● = Donor A, □ = Donor D [Heparin]: Low = 1.0 U/mL; High = 2.0 U/mL.

**Figure A9.** *In vitro* Case Study 1 platelet data worked up according to (6) showing % relative to the No material control platelet counts. Note that only Glass vs. PE vs. No Material is shown. ● = Donor C Run 1, ■ = Donor C Run 2, **○** = Donor D Run 1, □ = Donor D Run 2. [Heparin]: Low = 0.6 U/mL; High = 1.0 U/mL

**Figure A10.** *In vitro* Case Study 2 platelet data worked up according to (6) showing % relative to the No material control platelet counts. Note that only Glass vs. PE vs. No Material is shown. ● = Donor A Run 1, ■ = Donor A Run 2, **○** = Donor D Run 1, □ = Donor D Run 2. [Heparin]: Low = 0.6 U/mL; High = 1.0 U/mL.
