# Supplementary material for: In vitro methodology for medical device material thrombogenicity assessments: A use condition and bioanalytical proof‐of‐concept approach
Source: J Biomed Mater Res B Appl Biomater. 2020 Sep 14;109(3):358–76. doi: 10.1002/jbm.b.34705 (PMC7821245; doi:10.1002/jbm.b.34705)
Supplement: Supplementary file 1 — Appendix S1: Supporting Information. [file JBM-109-358-s001.zip › JBMB_34705_Table A3.docx]

| **Comparisons of Interest**  **Case Study 1**  (ER=6.0cm^2^/L) | Heat Map of Significant Means Comparisons | | | | | | | | |
| --- | --- | --- | --- | --- | --- | --- | --- | --- | --- |
|  | Donor C | | | Donor D | | | Donors C&D | | |
|  | TAT | βTG | Plts* | TAT | βTG | Plts* | TAT | βTG | Plts* |
| [Heparin] = Low | | | | | | | | | |
| Glass > No Material* |  |  |  |  |  |  |  |  |  |
| Glass > LMCD-1, Test-1* |  |  |  |  |  |  |  |  |  |
| LMCD-1 > Test-1* |  |  |  |  |  |  |  |  |  |
| Test-1 > No Material* |  |  |  |  |  |  |  |  |  |
| [Heparin] = High | | | | | | | | | |
| Glass > No Material* |  |  |  |  |  |  |  |  |  |
| Glass > LMCD-1, Test-1* |  |  |  |  |  |  |  |  |  |
| LMCD-1 > Test-1* |  |  |  |  |  |  |  |  |  |
| Test-1 > No Material* |  |  |  |  |  |  |  |  |  |

*****For the various comparisons involving platelet counts, the ‘>’ sign is replaced with ‘<’, given that platelet count generally decreases with increase in material thrombogenicity.
