# Supplementary material for: In vitro methodology for medical device material thrombogenicity assessments: A use condition and bioanalytical proof‐of‐concept approach
Source: J Biomed Mater Res B Appl Biomater. 2020 Sep 14;109(3):358–76. doi: 10.1002/jbm.b.34705 (PMC7821245; doi:10.1002/jbm.b.34705)
Supplement: Supplementary file 1 — Appendix S1: Supporting Information. [file JBM-109-358-s001.zip › JBMB_34705_Table A6.docx]

| **Comparisons of Interest**  **Case Study 5**  (ER=6.0 cm^2^/mL) | Heat Map of Significant Means Comparisons | | | | | |
| --- | --- | --- | --- | --- | --- | --- |
|  | Tube Model (VF) | | | Loop Model (SDF) | | |
|  | Measurement | | | Measurement | | |
|  | TAT | F1.2 | βTG | TAT | F1.2 | βTG |
| [Heparin] = Low | | | | | | |
| Glass > No Material |  |  |  |  |  |  |
| Glass > PE |  |  |  |  |  |  |
| Glass > LMCD-5 |  |  |  |  |  |  |
| Glass > Test-5 |  |  |  |  |  |  |
| PE > No Material |  |  |  |  |  |  |
| LMCD-5 > No Material |  |  |  |  |  |  |
| Test-5 > No Material |  |  |  |  |  |  |
| Test-5 > LMCD-5 |  |  |  |  |  |  |
| [Heparin] = High | | | | | | |
| Glass > No Material |  |  |  |  |  |  |
| Glass > PE |  |  |  |  |  | 0.06 |
| Glass > LMCD-5 |  |  |  |  |  |  |
| Glass > Test-5 | 0.07 |  |  |  |  |  |
| PE > No Material |  |  |  |  |  |  |
| LMCD-5 > No Material |  |  |  |  |  |  |
| Test-5 > No Material |  |  |  |  |  |  |
| Test-5 > LMCD-5 |  |  |  |  |  |  |
