# Supplementary figures and images for: In vitro methodology for medical device material thrombogenicity assessments: A use condition and bioanalytical proof‐of‐concept approach
Source: J Biomed Mater Res B Appl Biomater. 2020 Sep 14;109(3):358–76. doi: 10.1002/jbm.b.34705 (PMC7821245; doi:10.1002/jbm.b.34705)

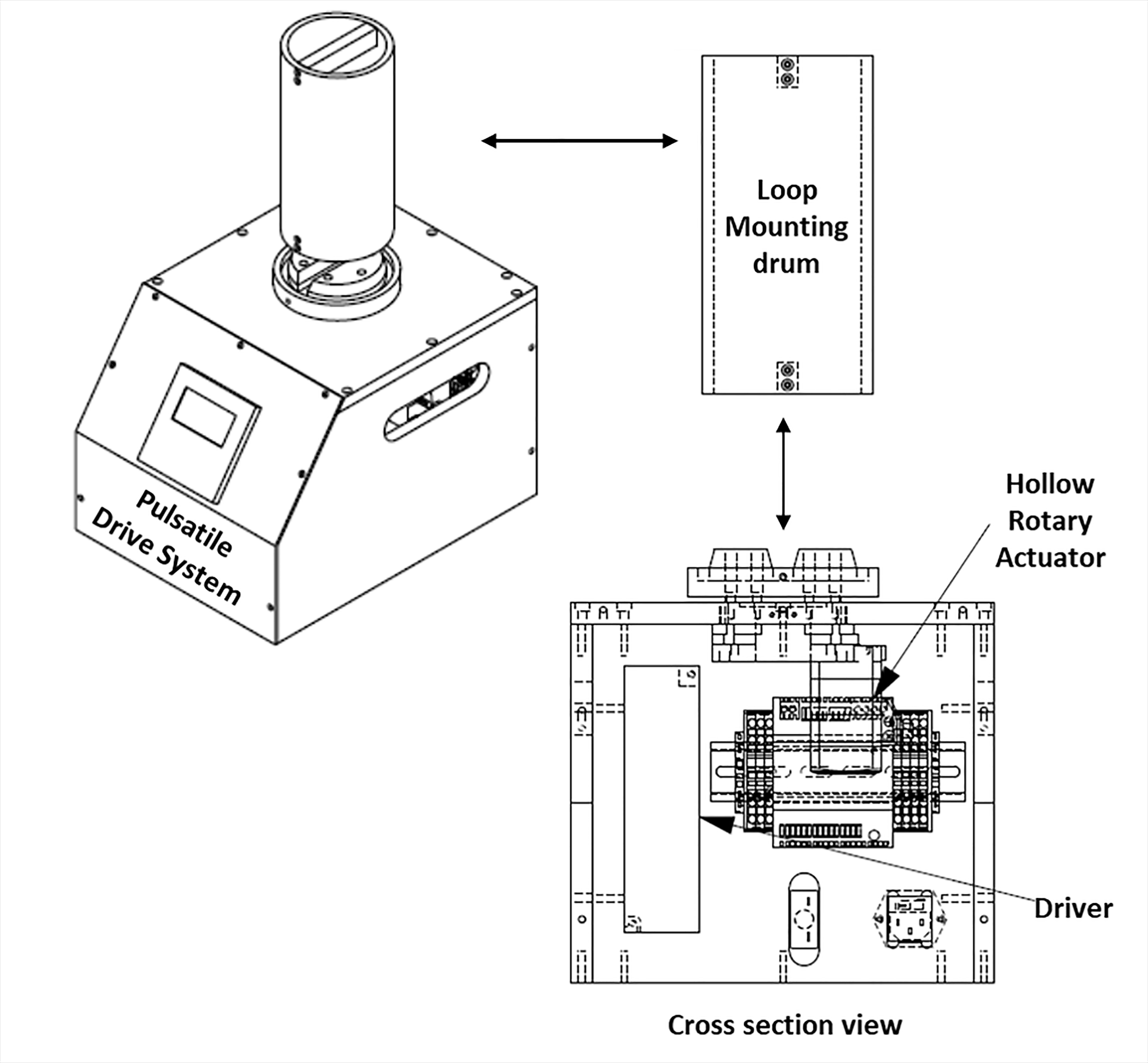

Supplement: Supplementary file 1 — Appendix S1: Supporting Information. [file JBM-109-358-s001.zip › JBMB_34705_Figure A1.tif]

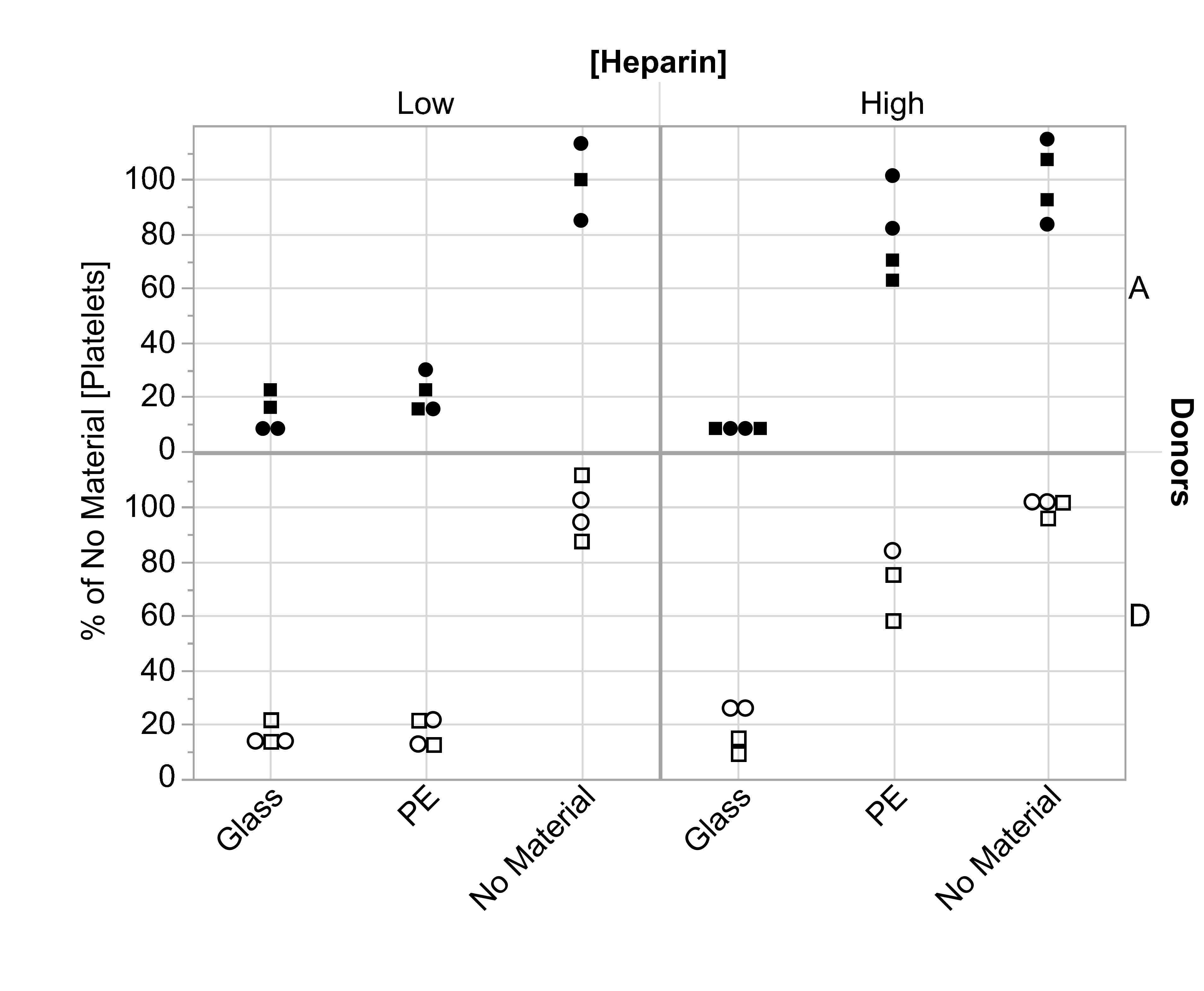

Supplement: Supplementary file 1 — Appendix S1: Supporting Information. [file JBM-109-358-s001.zip › JBMB_34705_Figure A10 600dpi.tif]

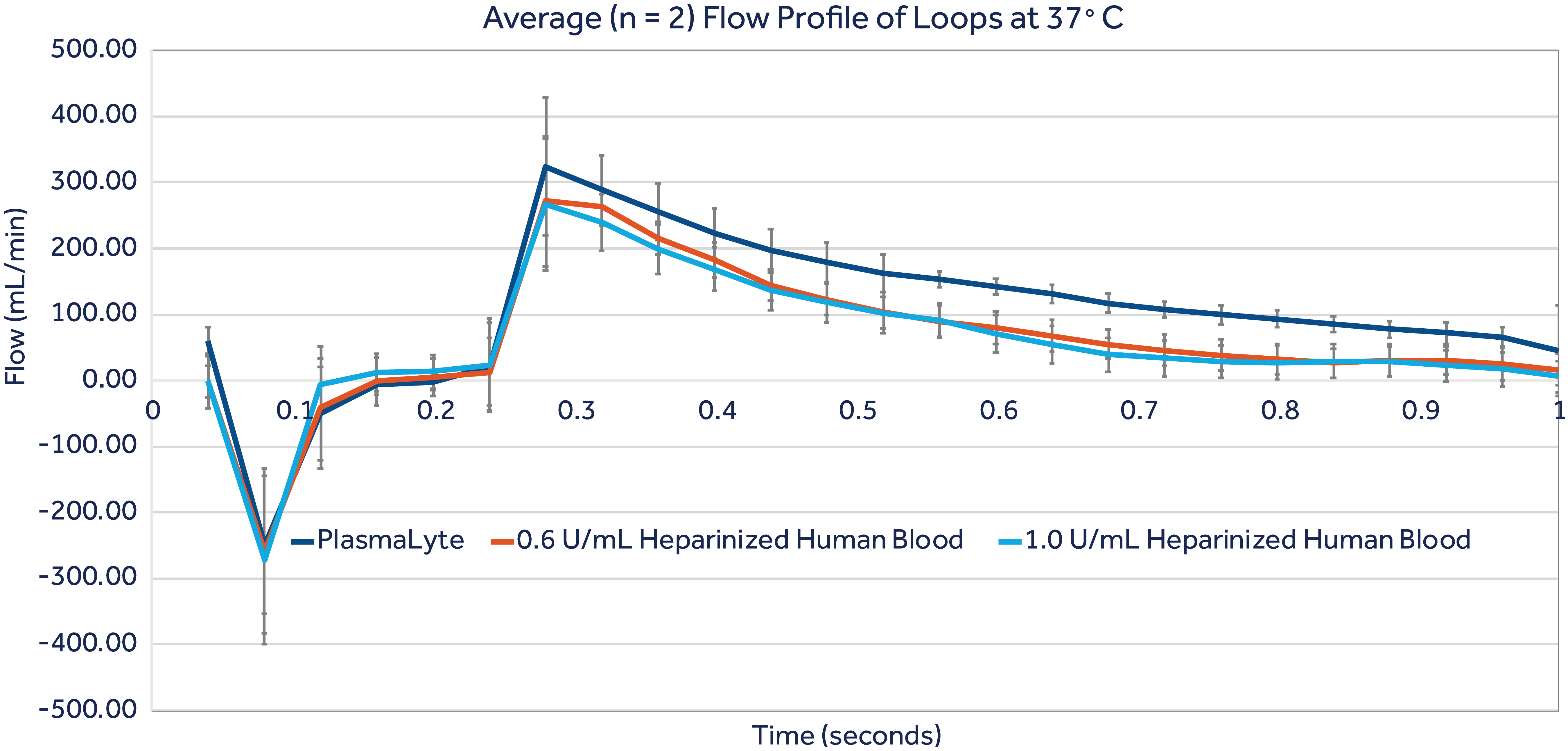

Supplement: Supplementary file 1 — Appendix S1: Supporting Information. [file JBM-109-358-s001.zip › JBMB_34705_Figure A2 .tif]

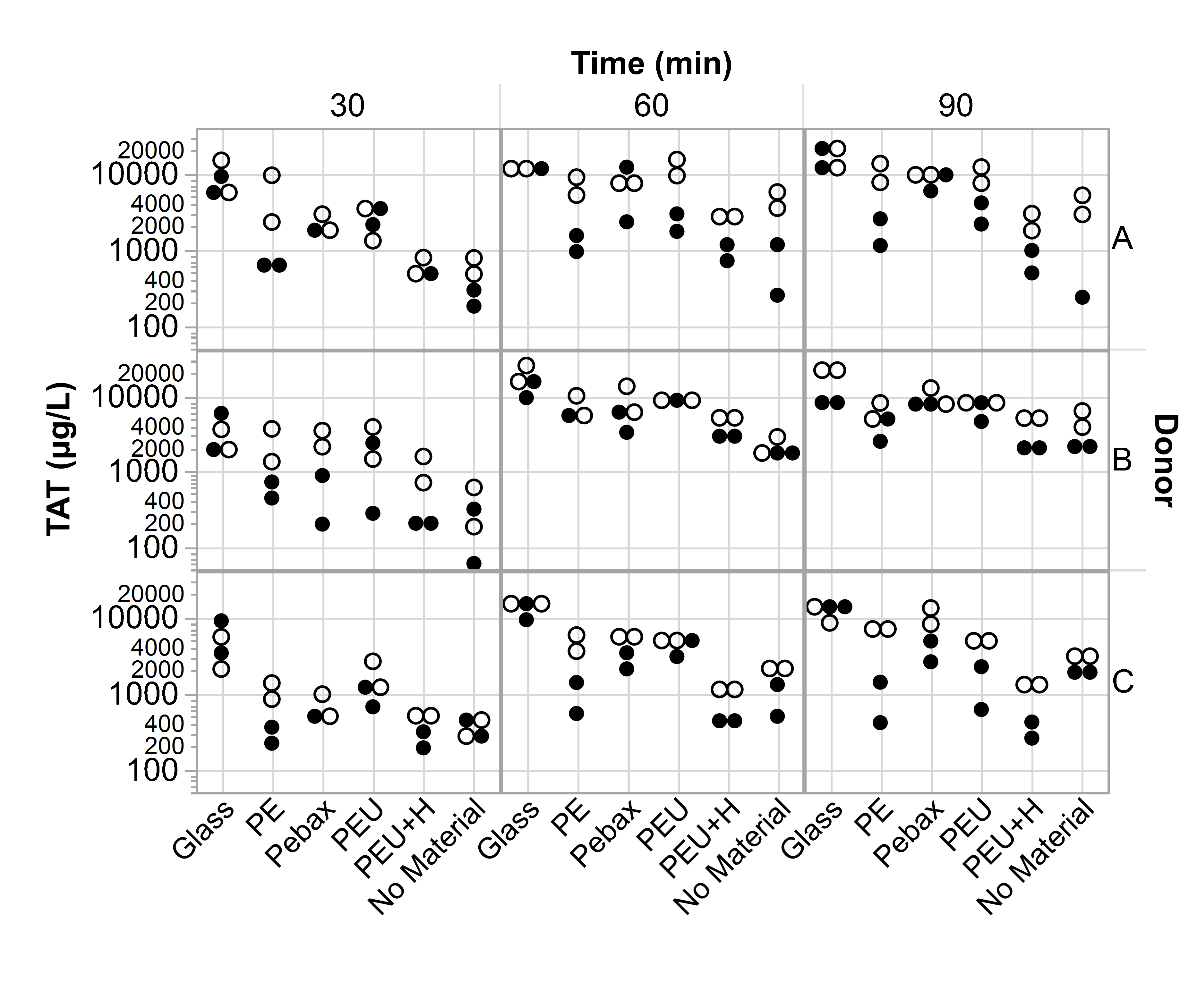

Supplement: Supplementary file 1 — Appendix S1: Supporting Information. [file JBM-109-358-s001.zip › JBMB_34705_Figure A3 A 600dpi.tif]

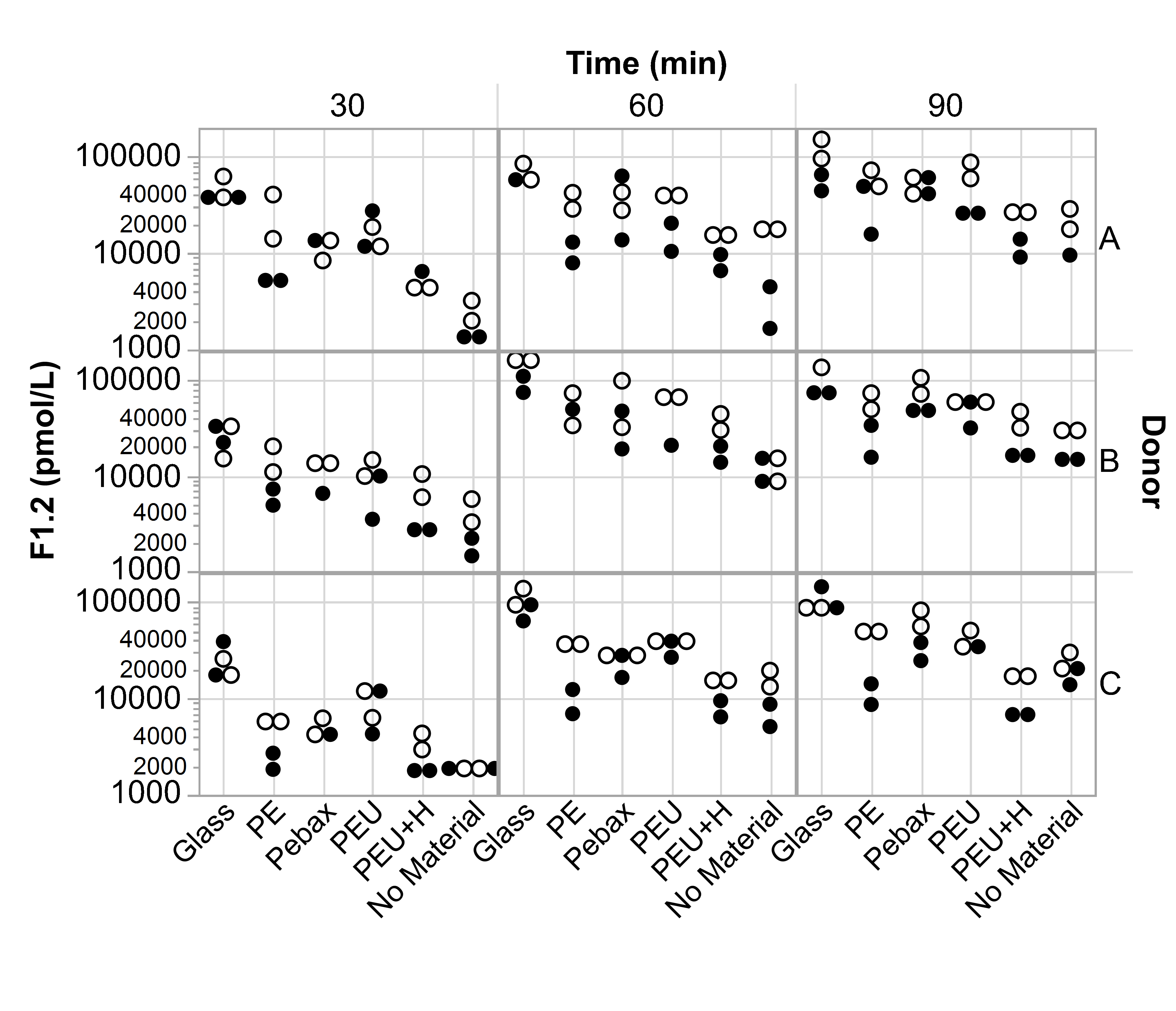

Supplement: Supplementary file 1 — Appendix S1: Supporting Information. [file JBM-109-358-s001.zip › JBMB_34705_Figure A3 B 600dpi.tif]

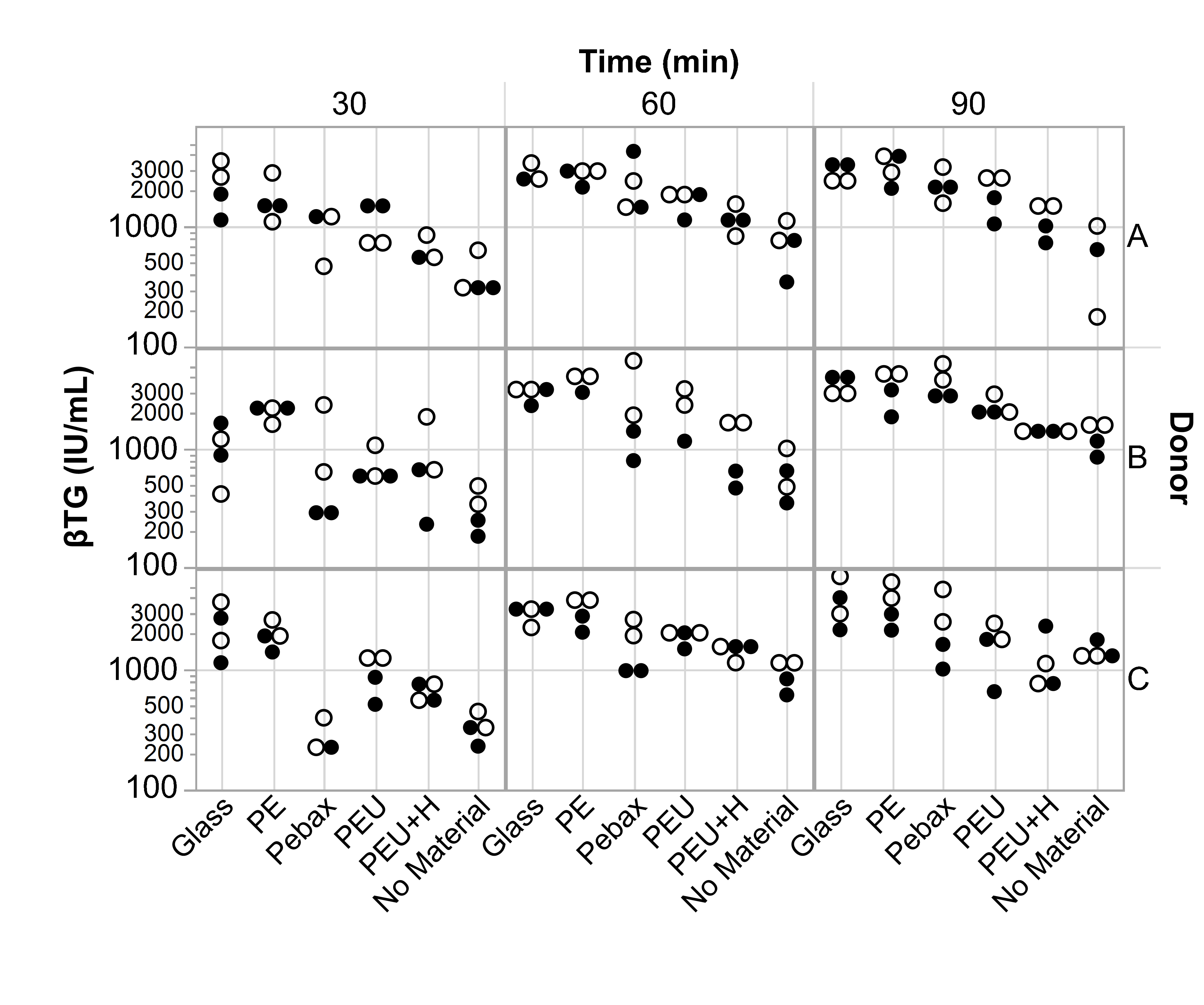

Supplement: Supplementary file 1 — Appendix S1: Supporting Information. [file JBM-109-358-s001.zip › JBMB_34705_Figure A3 C 600dpi.tif]

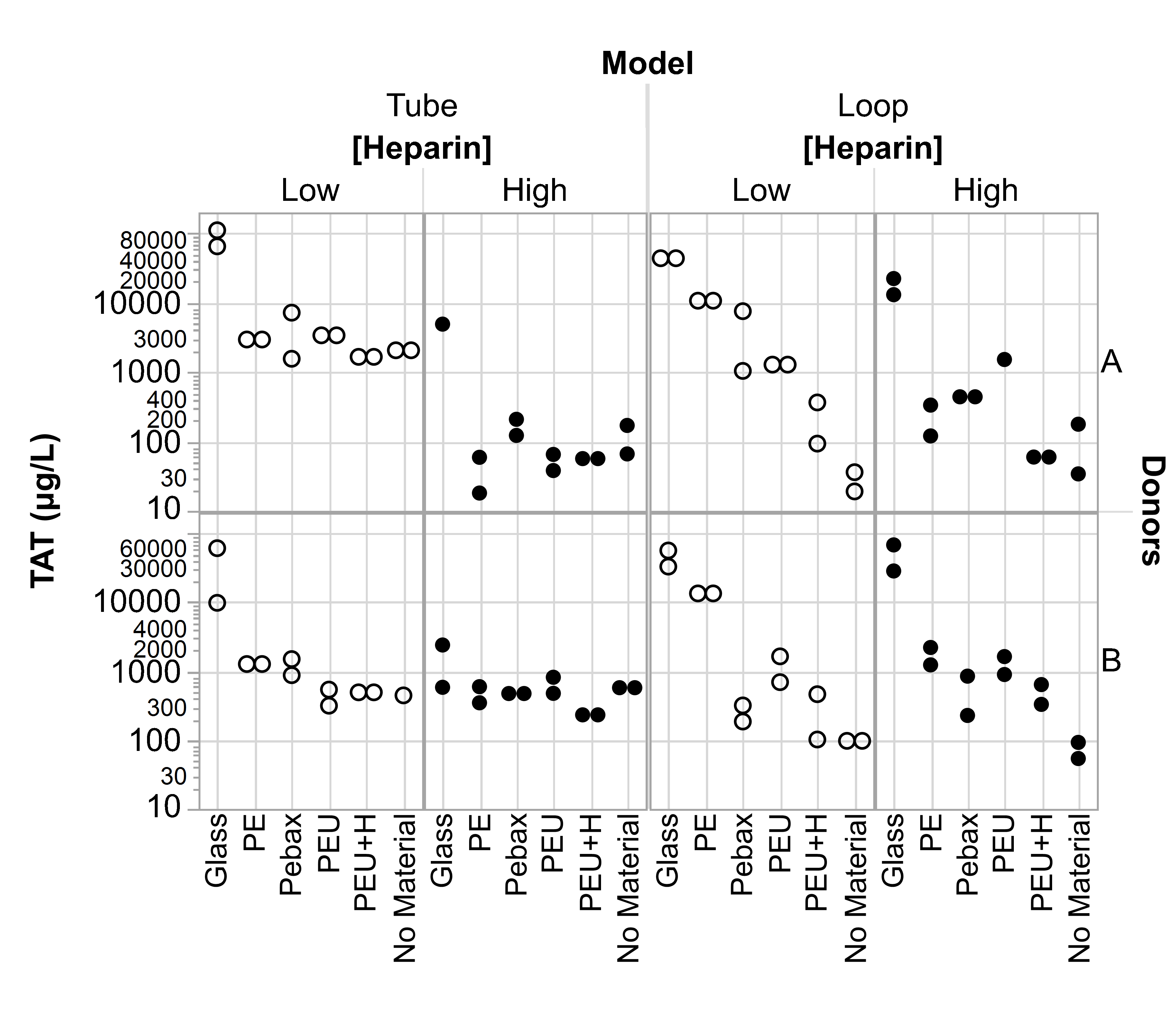

Supplement: Supplementary file 1 — Appendix S1: Supporting Information. [file JBM-109-358-s001.zip › JBMB_34705_Figure A4 A 600dpi.tif]

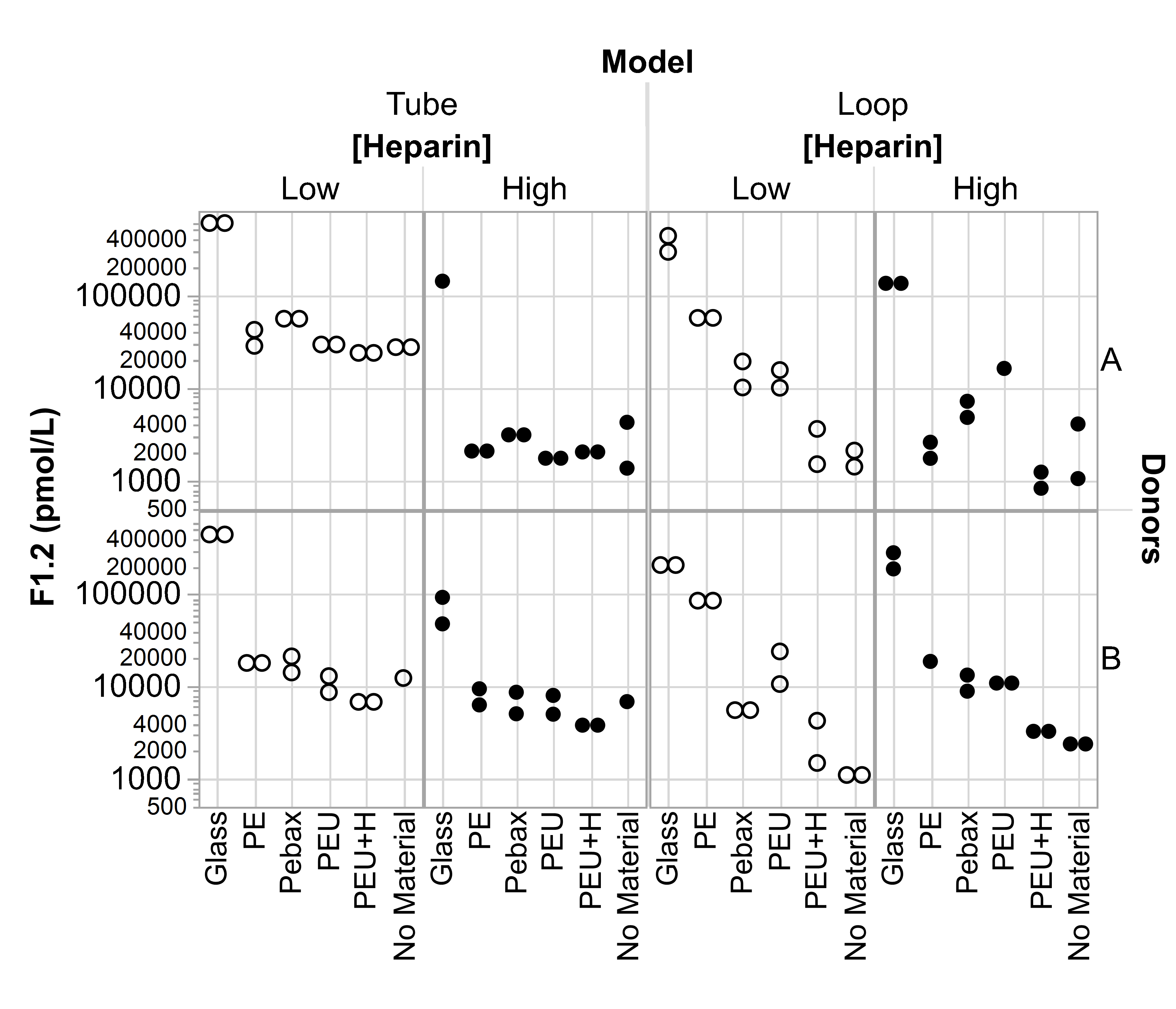

Supplement: Supplementary file 1 — Appendix S1: Supporting Information. [file JBM-109-358-s001.zip › JBMB_34705_Figure A4 B 600dpi.tif]

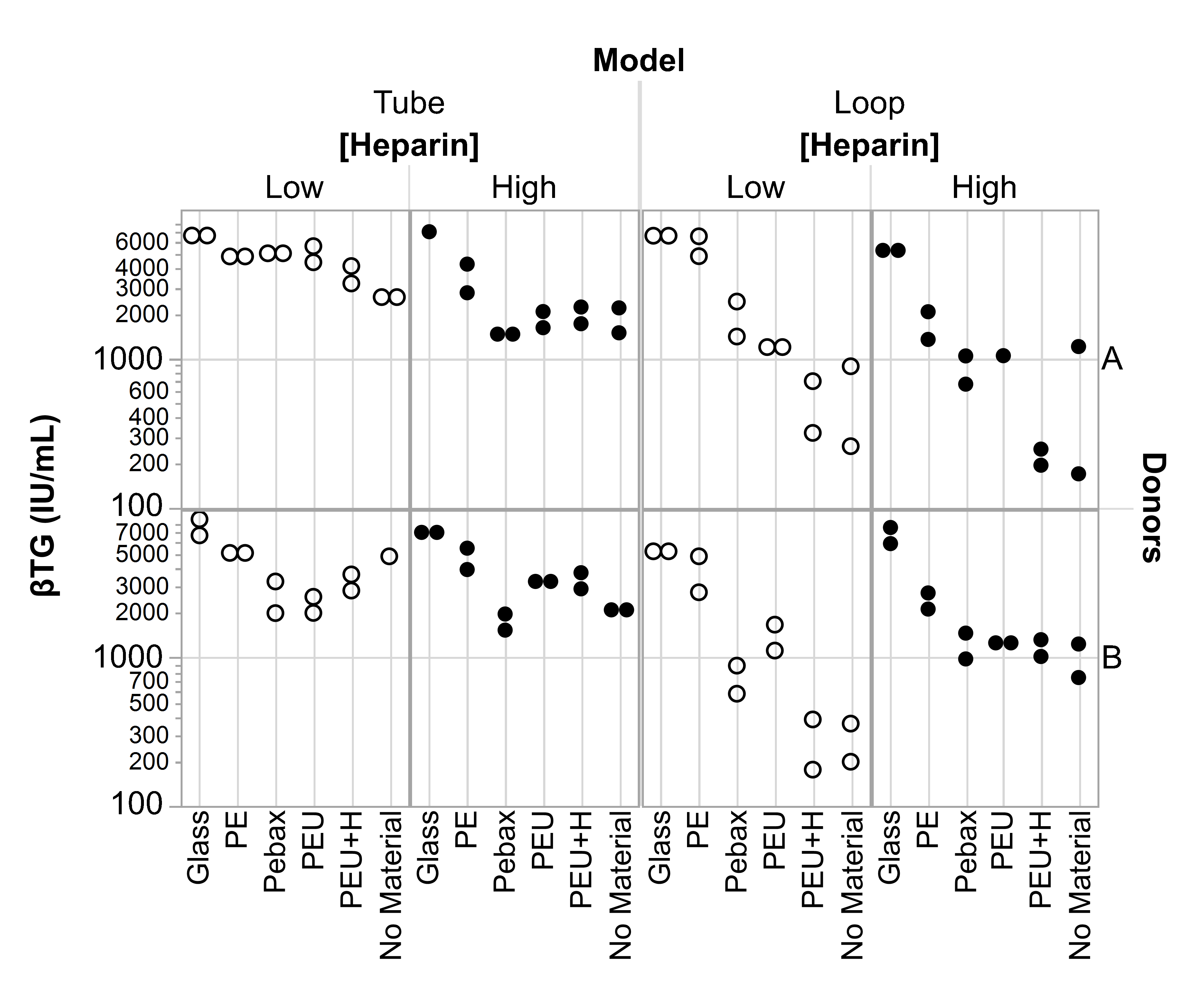

Supplement: Supplementary file 1 — Appendix S1: Supporting Information. [file JBM-109-358-s001.zip › JBMB_34705_Figure A4 C 600dpi.tif]

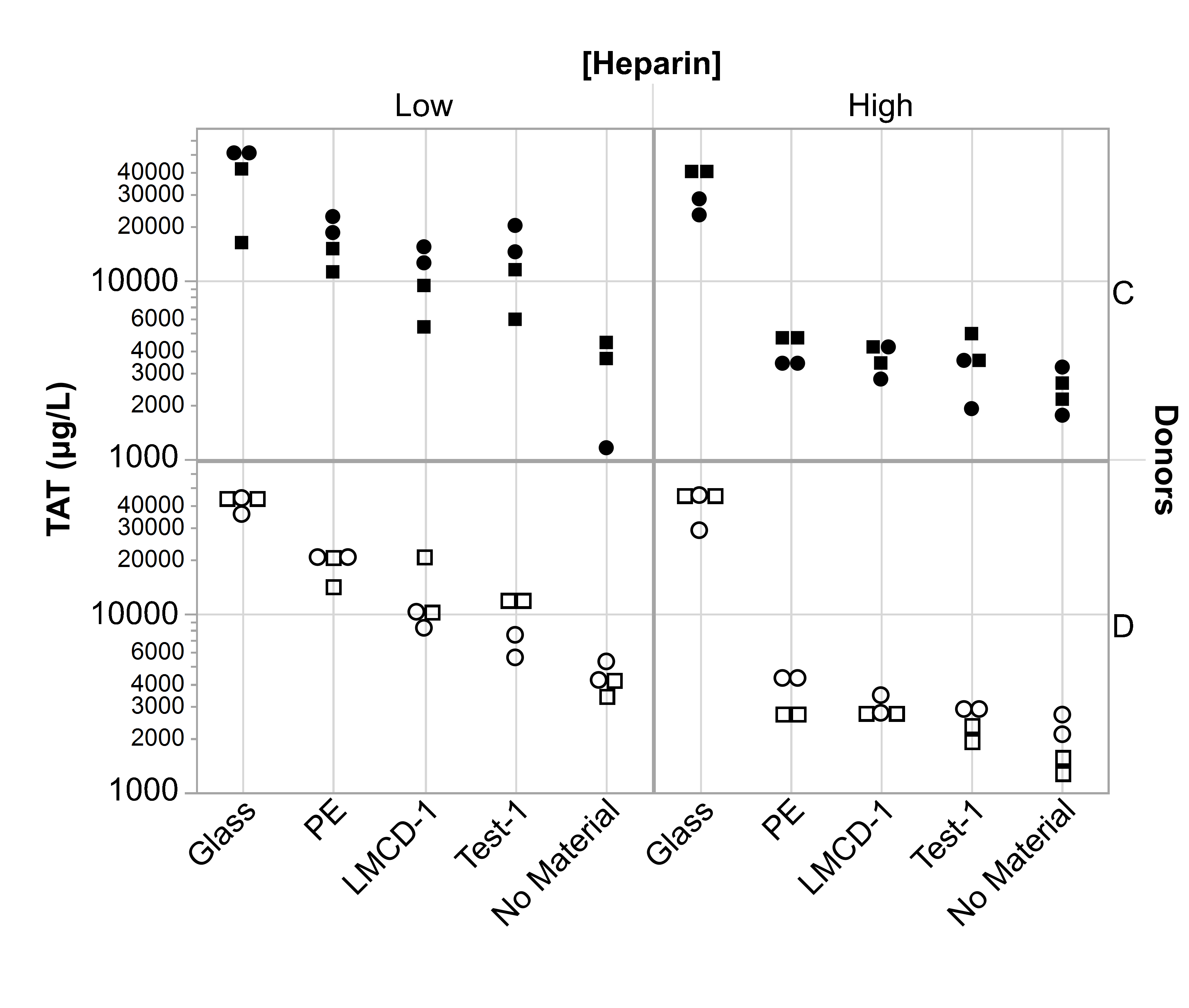

Supplement: Supplementary file 1 — Appendix S1: Supporting Information. [file JBM-109-358-s001.zip › JBMB_34705_Figure A5 A 600dpi.tif]

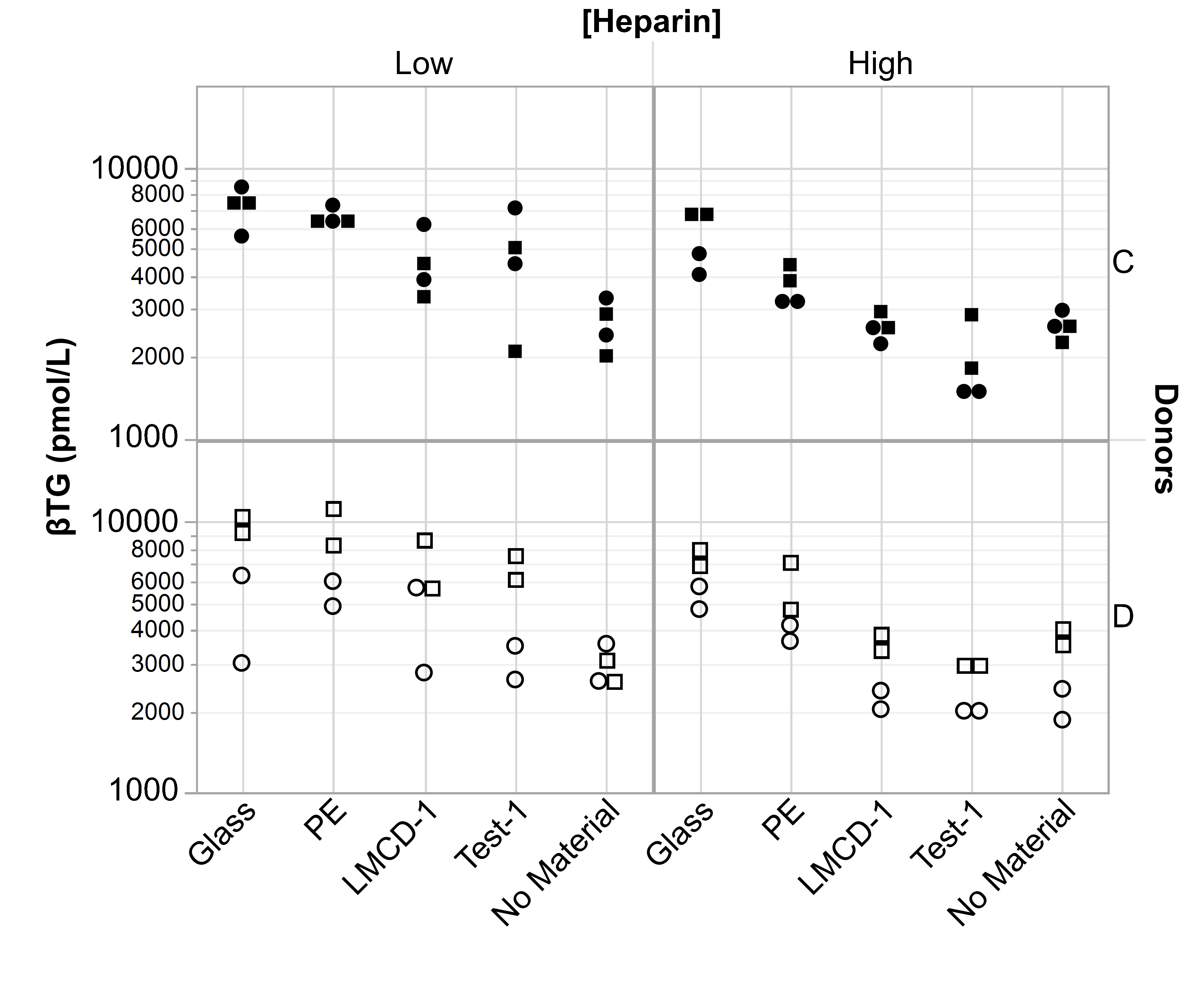

Supplement: Supplementary file 1 — Appendix S1: Supporting Information. [file JBM-109-358-s001.zip › JBMB_34705_Figure A5 B 600dpi.tif]

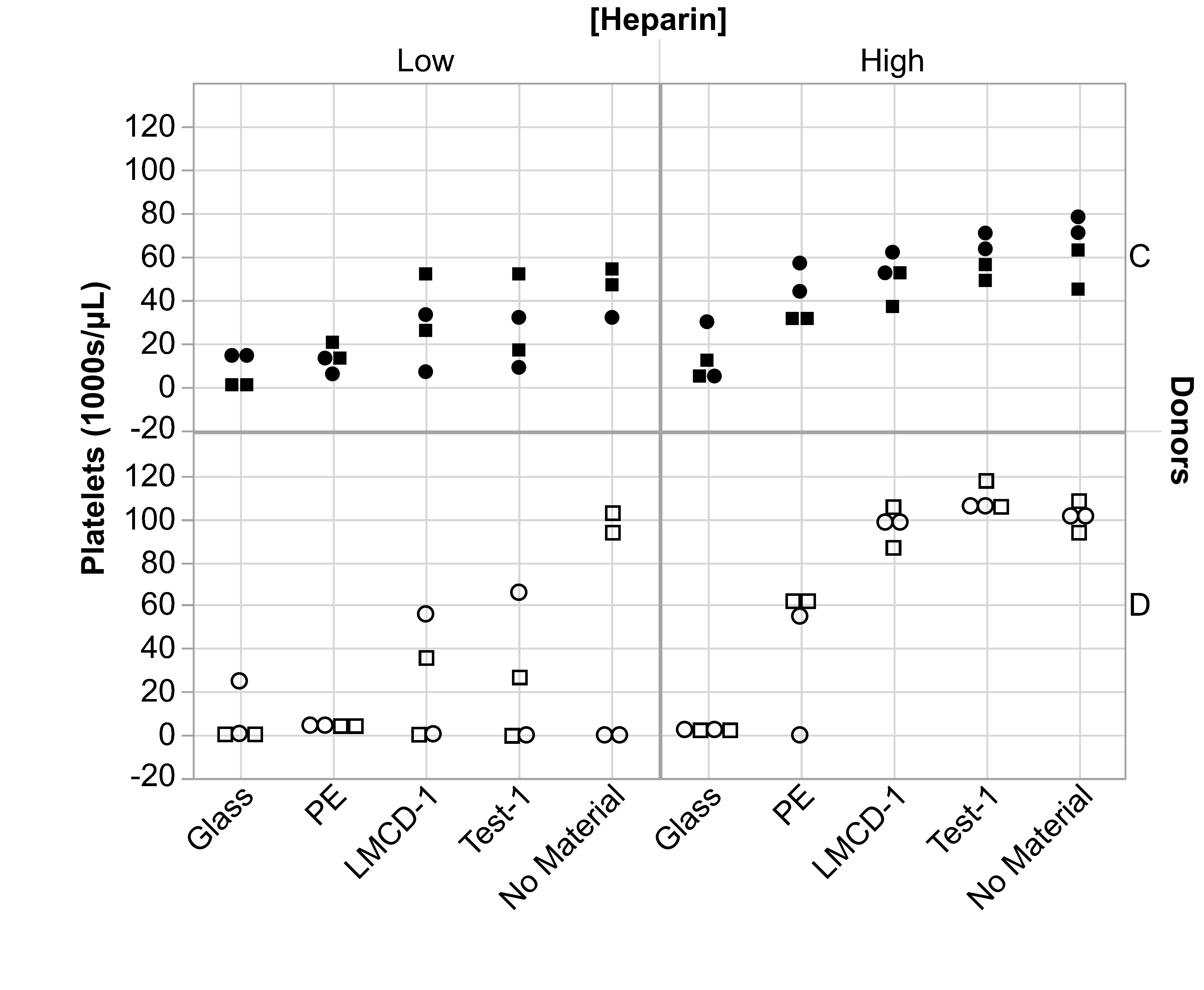

Supplement: Supplementary file 1 — Appendix S1: Supporting Information. [file JBM-109-358-s001.zip › JBMB_34705_Figure A5 C 600dpi.tif]

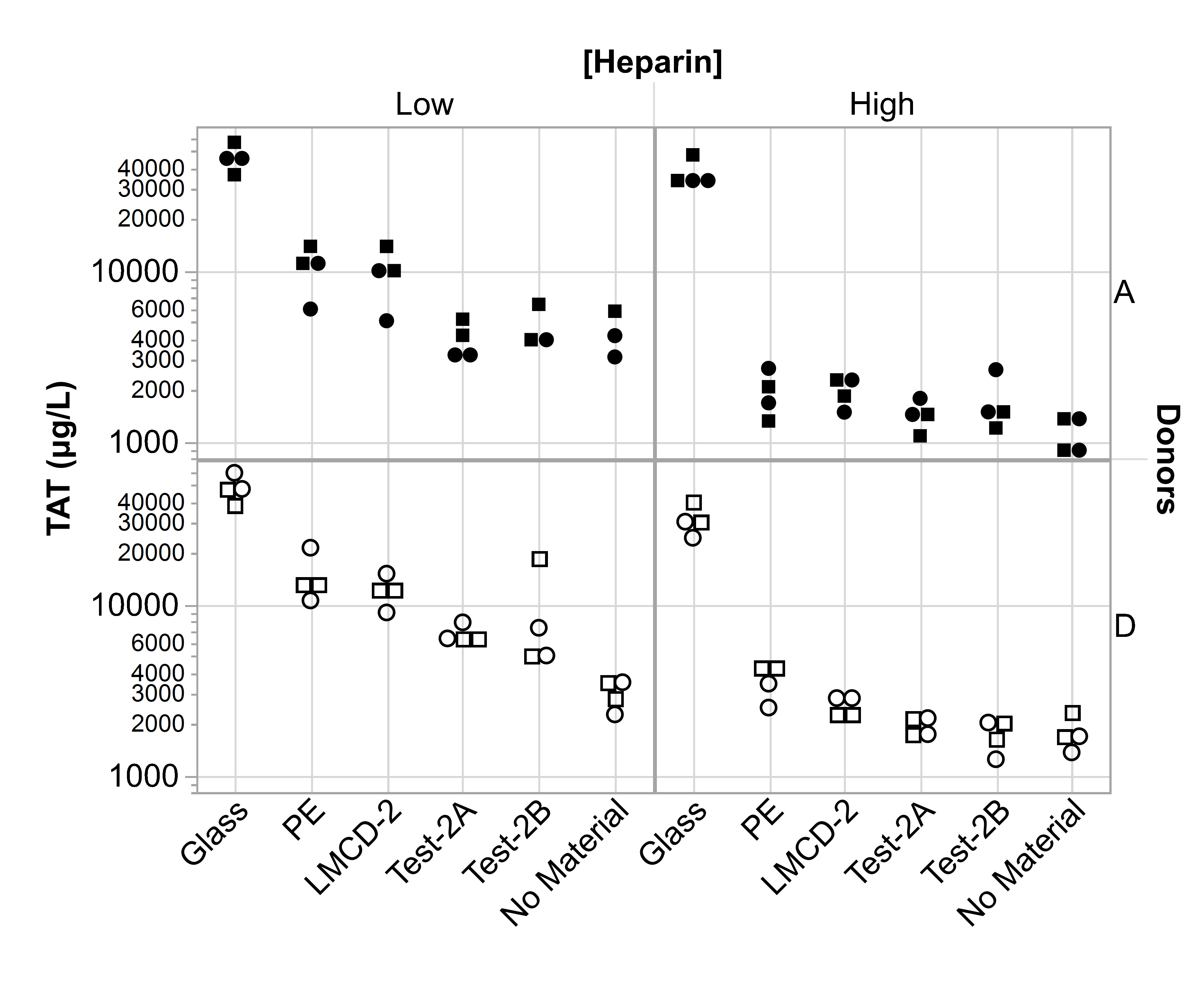

Supplement: Supplementary file 1 — Appendix S1: Supporting Information. [file JBM-109-358-s001.zip › JBMB_34705_Figure A6 A 600dpi.tif]

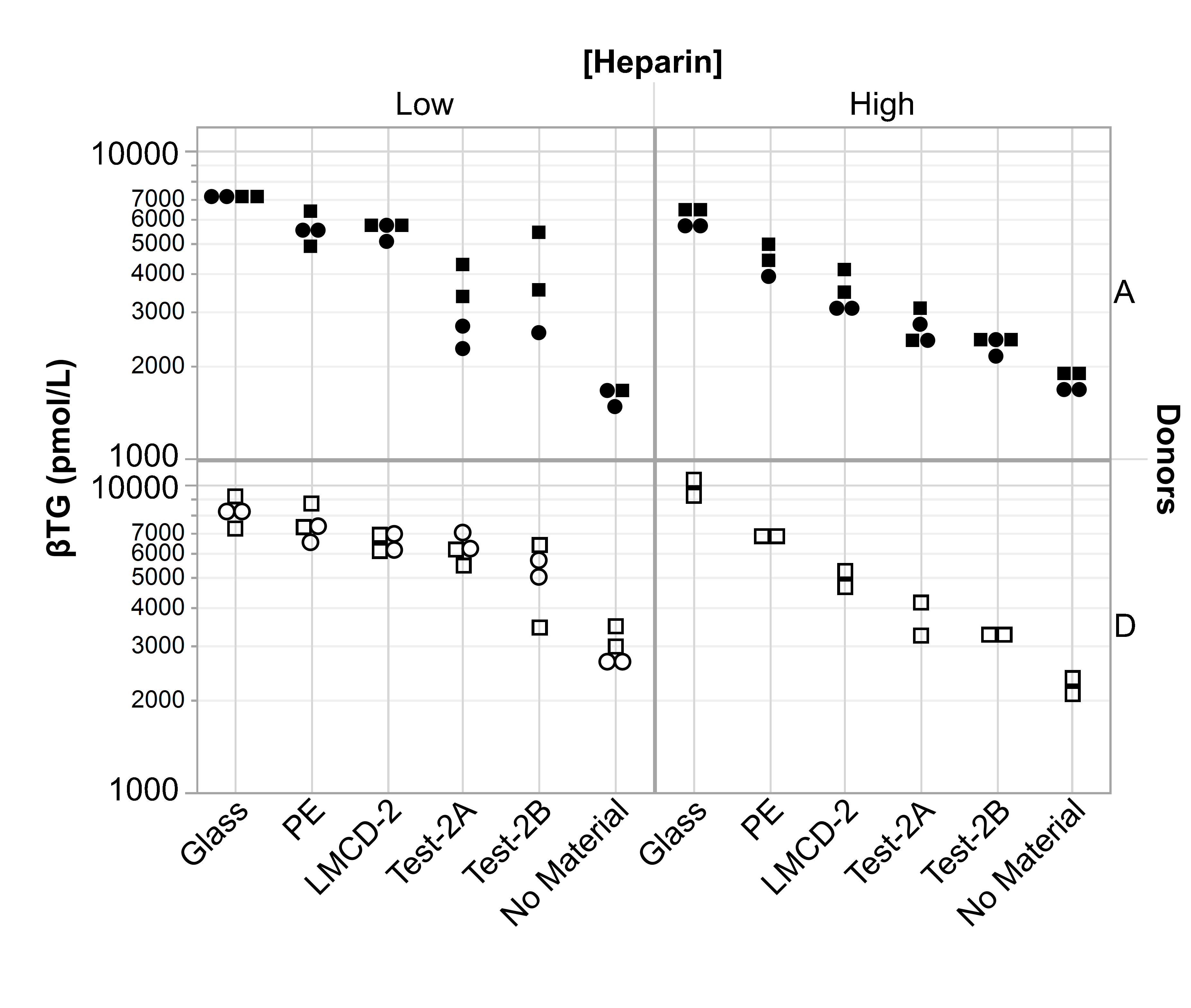

Supplement: Supplementary file 1 — Appendix S1: Supporting Information. [file JBM-109-358-s001.zip › JBMB_34705_Figure A6 B 600dpi.tif]

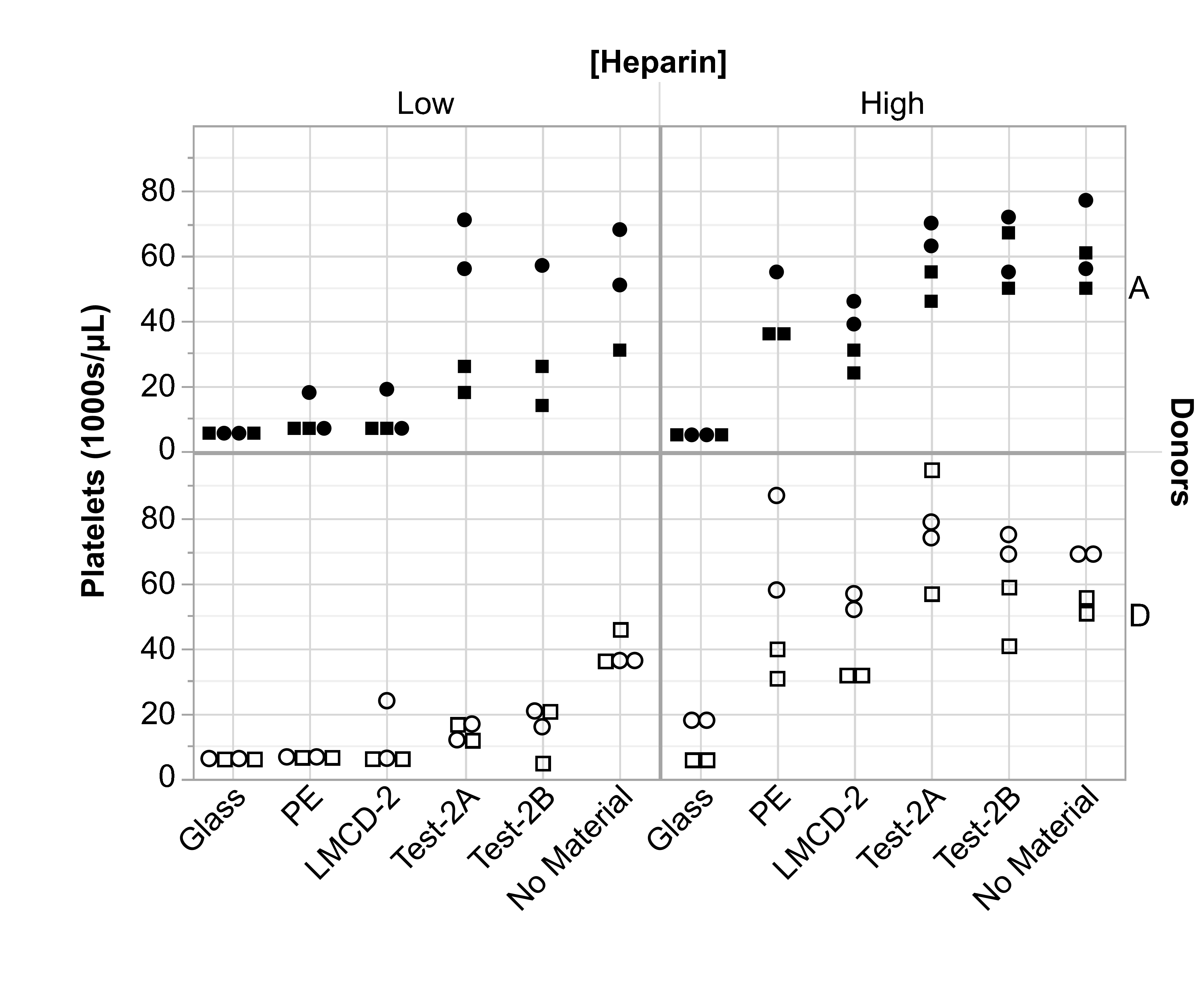

Supplement: Supplementary file 1 — Appendix S1: Supporting Information. [file JBM-109-358-s001.zip › JBMB_34705_Figure A6 C 600dpi.tif]

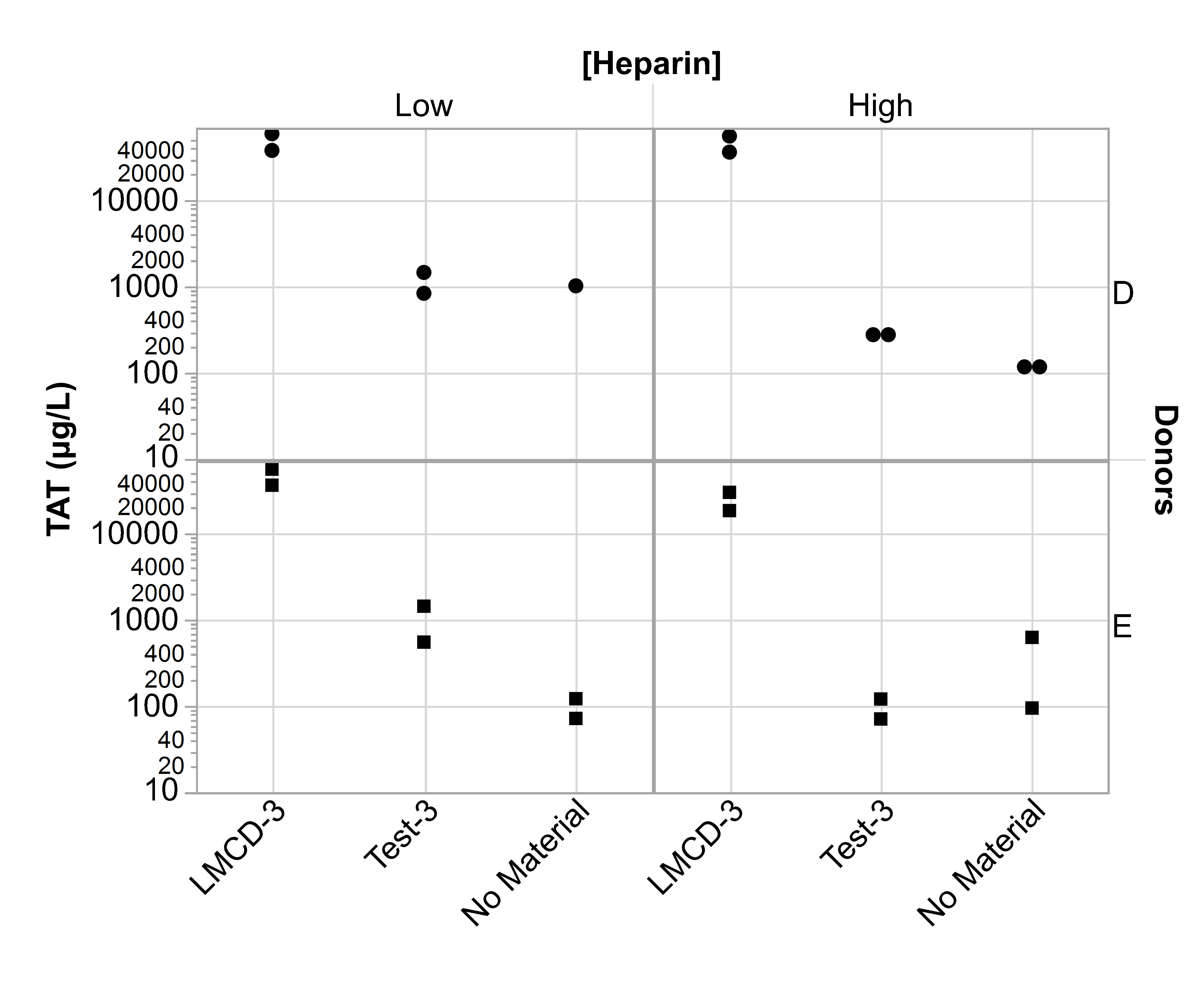

Supplement: Supplementary file 1 — Appendix S1: Supporting Information. [file JBM-109-358-s001.zip › JBMB_34705_Figure A7 A 600dpi.tif]

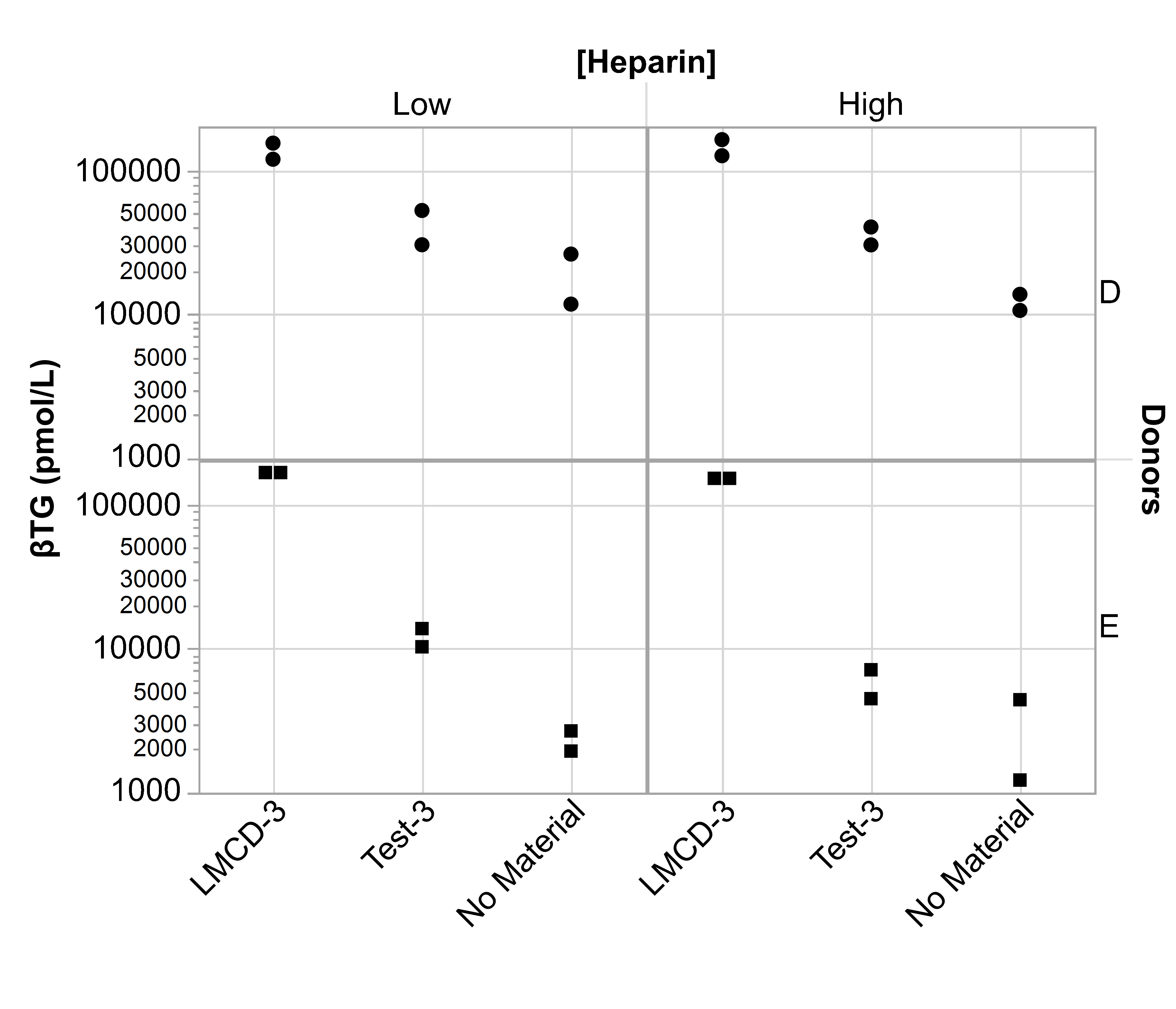

Supplement: Supplementary file 1 — Appendix S1: Supporting Information. [file JBM-109-358-s001.zip › JBMB_34705_Figure A7 B 600dpi.tif]

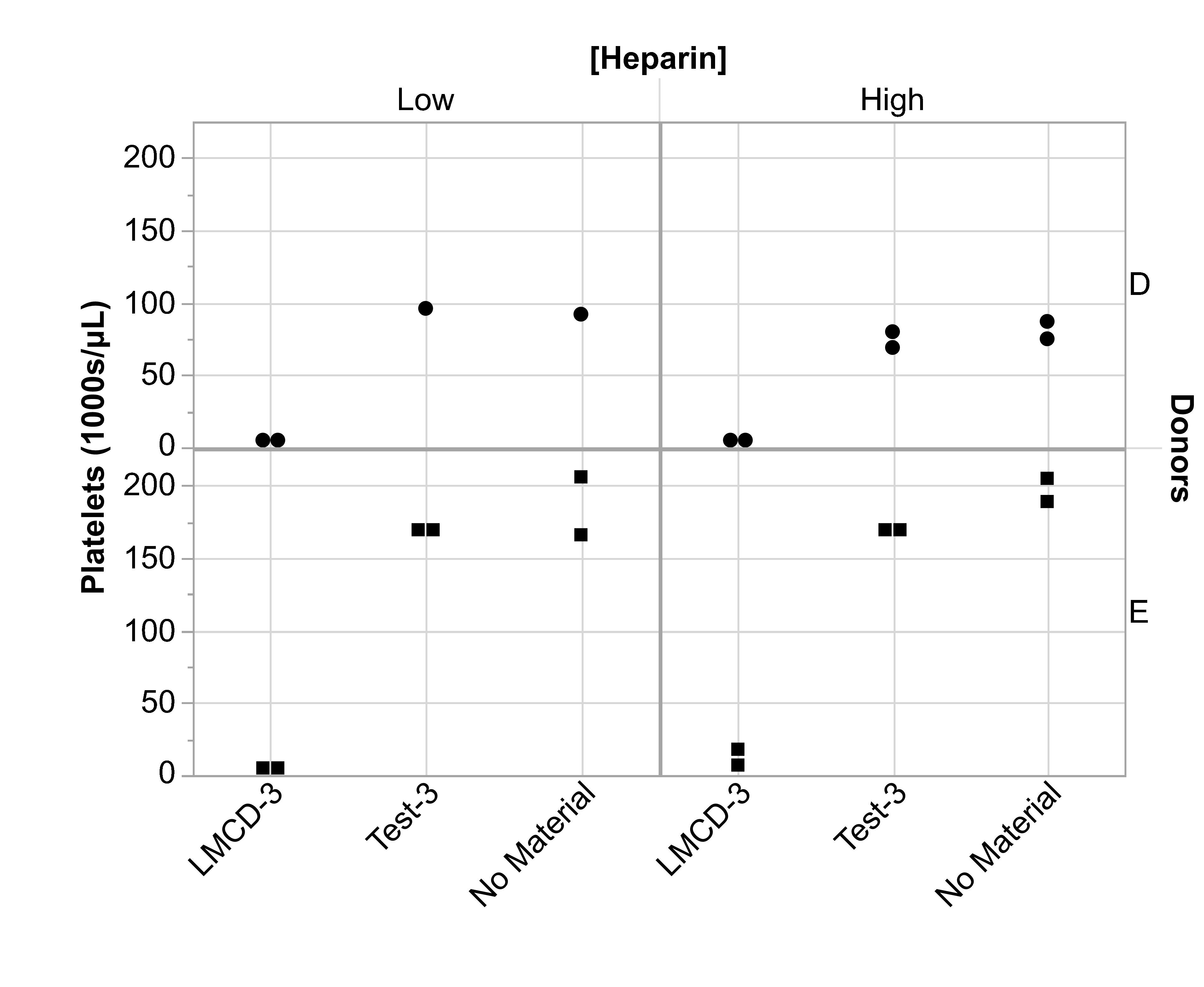

Supplement: Supplementary file 1 — Appendix S1: Supporting Information. [file JBM-109-358-s001.zip › JBMB_34705_Figure A7 C 600dpi.tif]

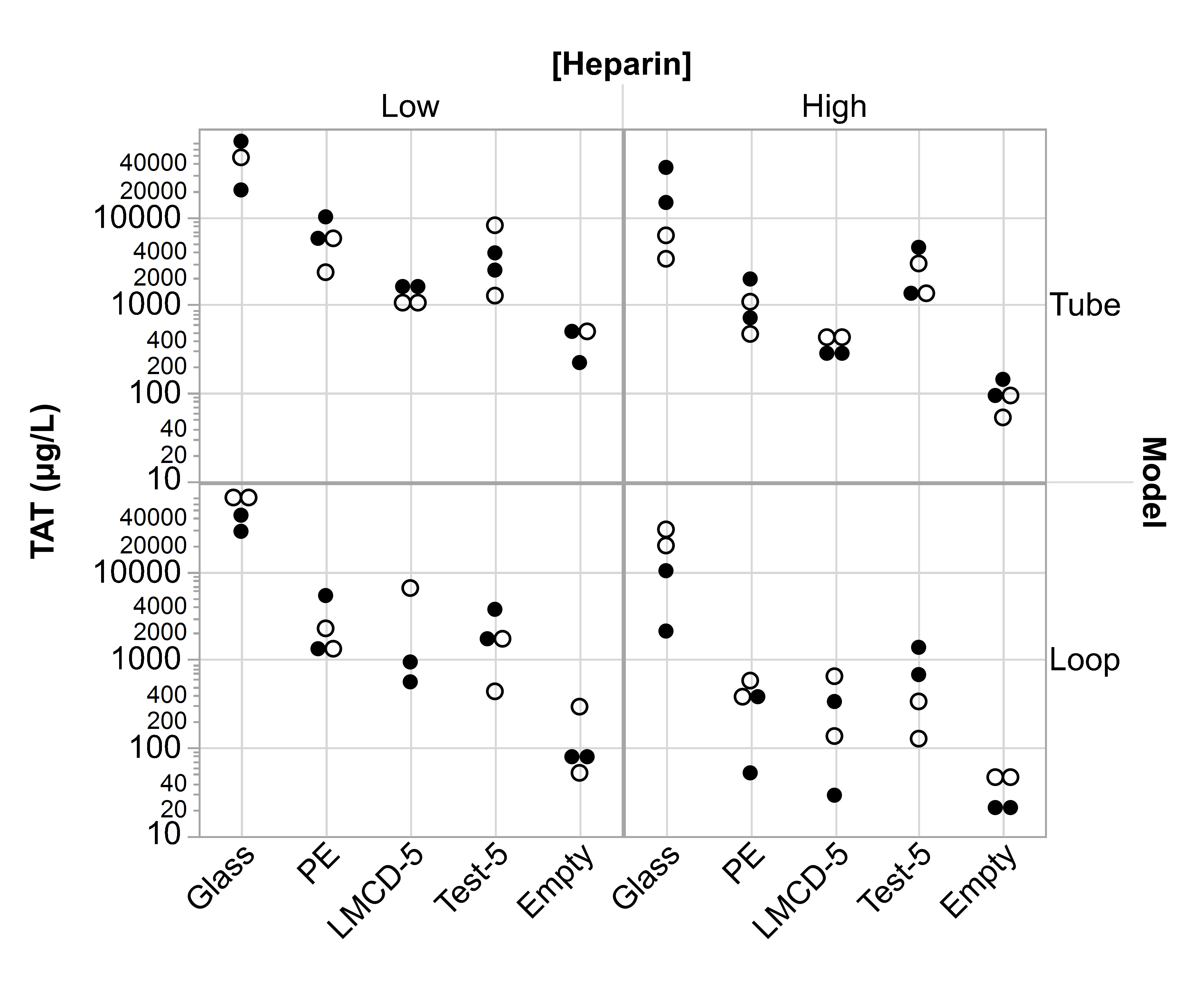

Supplement: Supplementary file 1 — Appendix S1: Supporting Information. [file JBM-109-358-s001.zip › JBMB_34705_Figure A8 A 600dpi.tif]

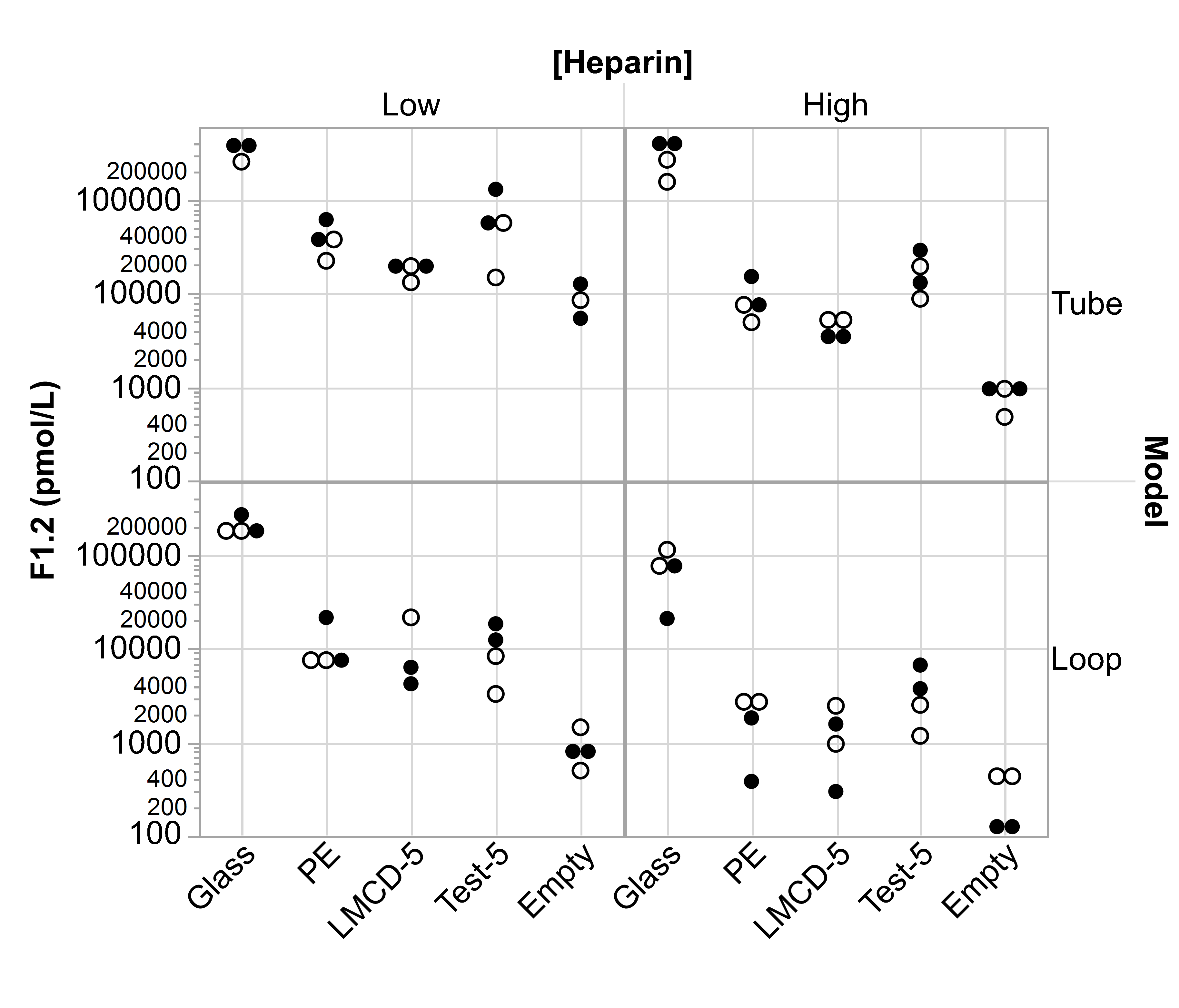

Supplement: Supplementary file 1 — Appendix S1: Supporting Information. [file JBM-109-358-s001.zip › JBMB_34705_Figure A8 B 600dpi.tif]

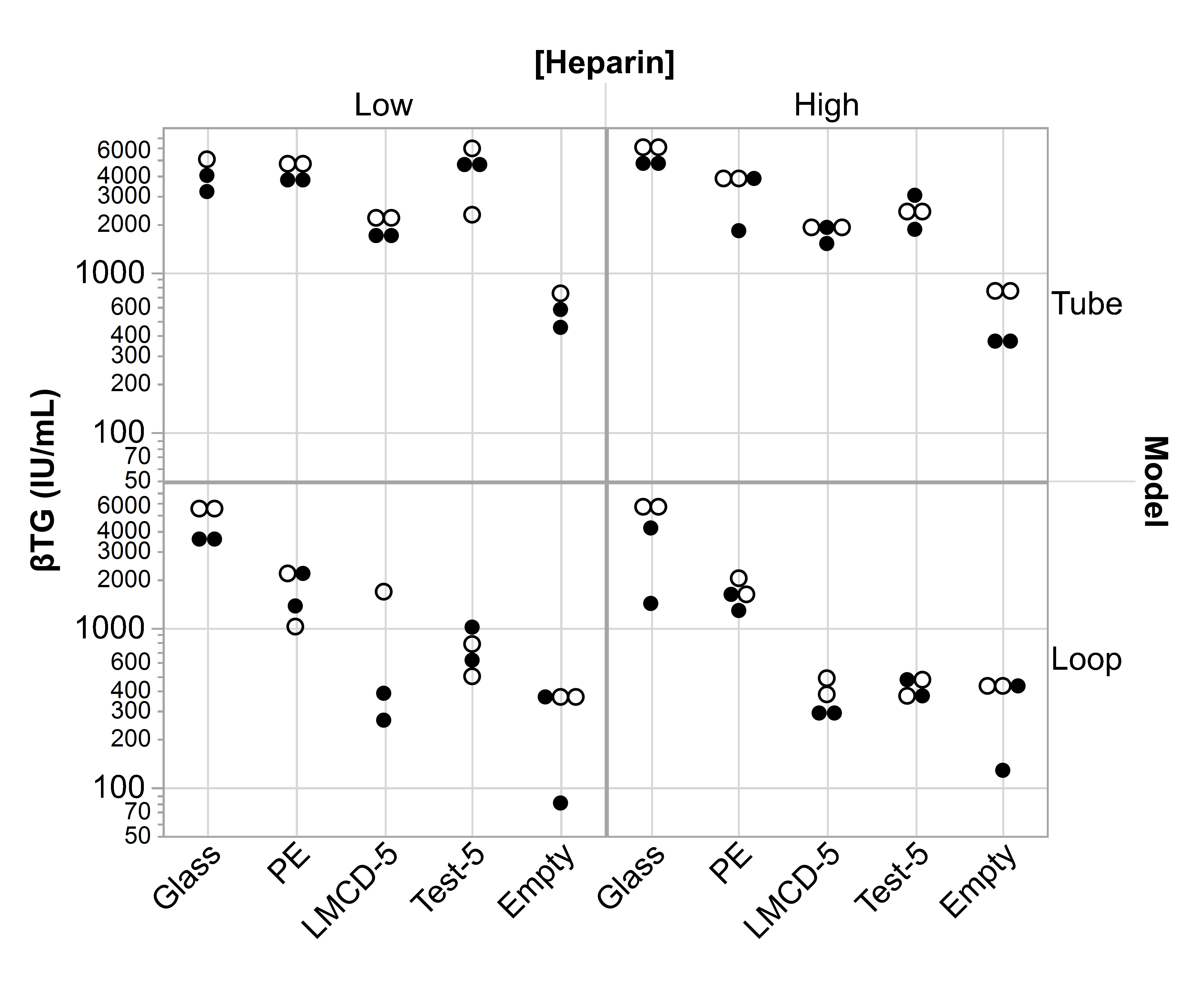

Supplement: Supplementary file 1 — Appendix S1: Supporting Information. [file JBM-109-358-s001.zip › JBMB_34705_Figure A8 C 600dpi.tif]

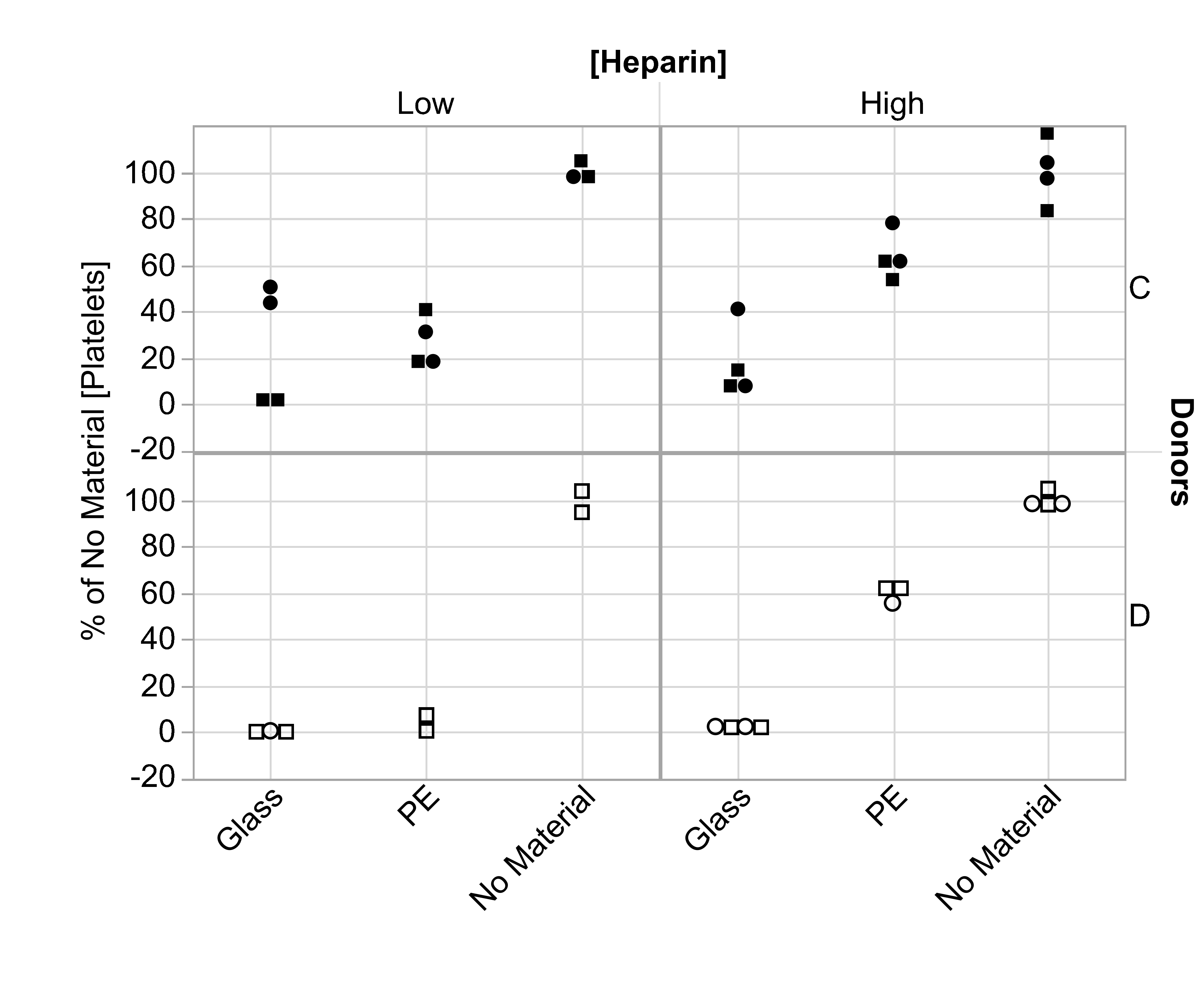

Supplement: Supplementary file 1 — Appendix S1: Supporting Information. [file JBM-109-358-s001.zip › JBMB_34705_Figure A9 600dpi.tif]
